# Synthesis and Antioxidant/Anti-Inflammatory Activity of 3-Arylphthalides

**DOI:** 10.3390/ph15050588

**Published:** 2022-05-10

**Authors:** María J. Ortega, Belén Parra-Torrejón, Fátima Cano-Cano, Laura Gómez-Jaramillo, M. Carmen González-Montelongo, Eva Zubía

**Affiliations:** 1 Departamento de Química Orgánica, Facultad de Ciencias del Mar y Ambientales, Universidad de Cádiz, Puerto Real, 11510 Cádiz, Spain; belen.parra@uca.es (B.P.-T.); eva.zubia@uca.es (E.Z.); 2 Unidad de Investigación, Instituto de Investigación e Innovación Biomédica de Cádiz (INiBICA), Hospital Universitario Puerta del Mar, Avda. Ana de Viya 21, 11009 Cádiz, Spain; canocano.fatima@gmail.com (F.C.-C.); laugomjar@gmail.com (L.G.-J.); mcmontel@gmail.com (M.C.G.-M.)

**Keywords:** phthalides, 3-arylphthalides, dehydrative coupling reaction, antioxidant, anti-inflammatory, cytokines

## Abstract

Phthalides are a group of compounds with relevant biological activities in different areas such as cytotoxicity, anti-stroke activity, neuroprotection, and inflammation, among others. In this study we designed and synthesized a series of 3-arylphthalide derivatives in order to identify their antioxidant and anti-inflammatory activities. The synthetic methodology was established in terms of atom and step economy through a dehydrative coupling reaction between 3-hydroxyphthalide and different properly functionalized arene rings. The evaluation of the antioxidant activity was performed by the ABTS assay and for the anti-inflammatory activity the inhibition of LPS-induced nitric oxide (NO) production in microglial cells Bv.2 and macrophage cells RAW 264.7 was measured. The synthesized compound 3-(2,4-dihydroxyphenyl)phthalide (**5a**) showed better antioxidant activity than the Trolox standard and caused strong inhibition of NO production in LPS-stimulated Bv.2 and RAW 264.7 cells. In addition, compound **5a** reduced the expression of the pro-inflammatory cytokines *Il1b* and *Il6* in RAW 264.7 cells. These results, which are the first account of the anti-inflammatory activity of 3-arylphthalides, suggest that compound **5a** could be a promising candidate for more advanced anti-inflammatory studies.

## 1. Introduction

**Figure 1:**
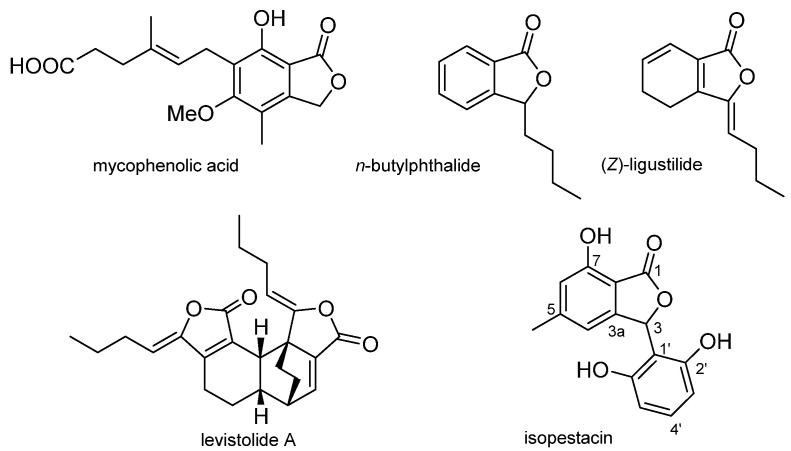
Representative examples of structural diversity of bioactive phthalides.

Naturally occurring phthalides are a relatively small group of metabolites produced by a variety of plants, including species worldwide used in traditional medicine, and also by fungi from terrestrial and marine environments [1]. Structurally, these natural products are characterized by the presence of a 1(*3H*)-isobenzofuranone nucleus (phthalide), the substitution patterns of which confer wide structural diversity on this family of compounds (Figure 1). Thus, the aromatic ring of the phthalide often contains hydroxy or alkoxy groups, although sugars and moieties of terpenoid or alkaloid origin may also be present. With regard to the γ-lactone ring, most natural phthalides are substituted at C-3, exhibiting alkyl chains, spirocycles, and aromatic rings either alone or as part of more complex substructures. In addition, compounds with the aromatic ring partially reduced and dimeric phthalides have also been described as natural products [1,2].

Phthalides have been the subject of numerous pharmacological studies which have shown a wide range of properties, such as cytotoxic, antifungal, antibacterial, anti-inflammatory and anti-stroke activities, among others, in this class of natural products [1,2,3]. Two remarkable compounds are mycophenolic acid and 3-*n*-butylphthalide (NBP) (Figure 1). Mycophenolic acid, first isolated from *Penicillium stoloniferum*, [4] is used as an immunosuppressive drug in organ transplantation [5]. NBP, firstly isolated from *Angelica sinenxis* Radix [6], was approved by the State Food and Drug Administration of China as drug for the treatment of ischemic stroke [7].

Over the last decades, the study of inflammatory processes has become a main area of research due to the close relationship between inflammation and the development of serious diseases such as certain types of cancer, inflammatory bowel disease, diabetes, and arthritis, among others [8,9]. Moreover, inflammation is related to oxidative stress since reactive oxygen species (ROS) are potent inflammatory mediators [10]. Inflammation is usually treated with non-steroidal anti-inflammatory drugs that, after a prolonged use, often lead to undesirable side effects [11]. Therefore, novel therapeutic agents with anti-inflammatory properties and minimal side effects are needed. In this context, various studies have described the anti-inflammatory activity of several natural phthalides. These studies have focused on (*Z*)-ligustilide (Figure 1) [12,13,14,15,16,17,18], cnidilide [19] and related compounds and dimers, as well as some semi-synthetic derivatives [12,18,20,21,22,23]. It is worth noting that all these compounds are phthalides bearing one or two alkyl residues at C-3, while no data on 3-aryl derivatives have been found in the literature.

On this basis, in the course of our research on anti-inflammatory natural products, we turned our attention towards phthalides bearing at C-3 an aromatic ring, such as isopestacin (Figure 1) [24]. As the first approach to exploring the anti-inflammatory potential of 3-arylphthalides, we undertook the synthesis of a series of phthalides substituted at C-3 with benzene rings bearing oxygenated, halogenated, and/or sulfur functionalization. In this regard, functional groups such as hydroxy or alkyloxy groups can exert an important influence on antioxidant activity [25], and sulfur-containing drugs have also exhibited good efficiency in several aspects, including antioxidant, anti-free-radical, and neuroprotective activities, among others [26]. Then, the synthesized compounds were tested in antioxidant and anti-inflammatory assays.

## 2. Results and Discussion

### 2.1. Synthesis of 3-Arylphthalides

The important role of phthalides, both in total synthesis and pharmaceutical chemistry, has attracted attention of chemists and several synthetic strategies have been developed [1,3,27,28,29]. The strategies first described were based preferentially on three synthetic aspects: the construction of the γ-lactone on a six carbon cycle, the construction of the benzene system on a pre-existing γ-lactone, or the concomitant formation of both rings, the benzene and the γ-lactone rings [3]. More recently, other strategies have been developed, searching either one-pot multicomponent reaction (MCRs) versions as powerful tools for the rapid assembly of molecules [30] or innovative C–H bond functionalization using simple and unactivated starting materials with the achievement of atom economy and molecular diversity [29].

**Figure 2:**
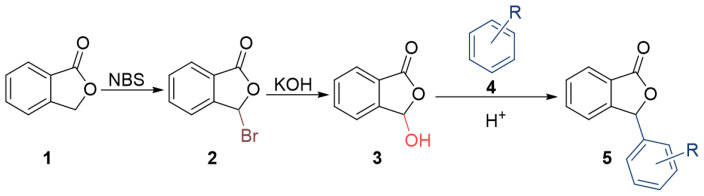
Synthesis of 3-arylphthalides.

The synthetic strategy herein, proposed to obtain 3-substituted phthalide derivatives, is established in terms of atom and step economy through a dehydrative coupling reaction in which a C–H and a C–OH bonds react to form a C–C bond with elimination of a water molecule [31]. In particular, the direct condensation of 3-hydroxyphthalide with different arene rings under acid catalysis is established. The synthetic route for the preparation of 3-arylphthalide derivatives is outlined in Figure 2.

**Figure 3:**
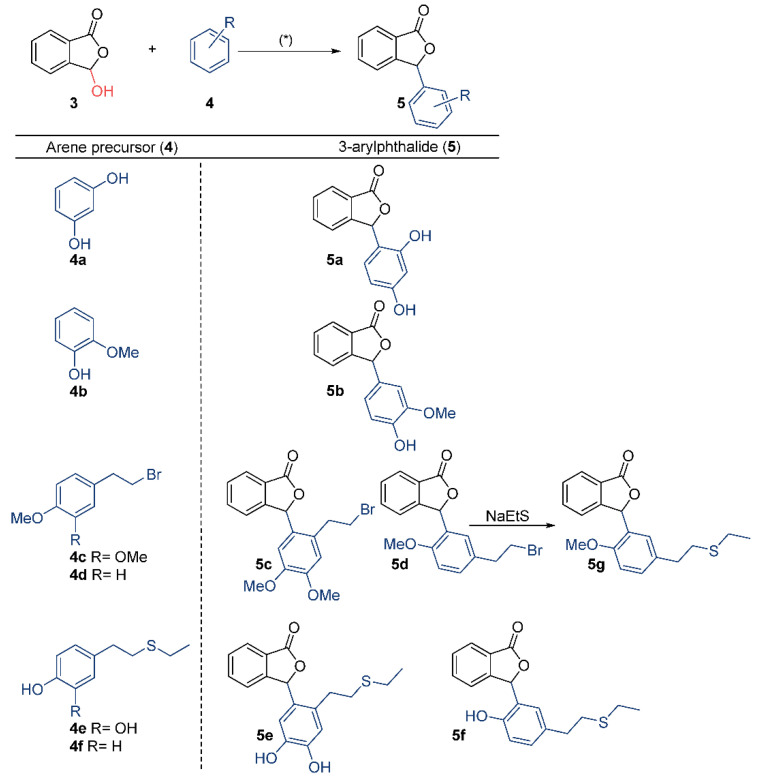
Synthesis of compounds **5a**–**5g**. (*) dioxane/H_2_O (1:4) + 5% HCl for **5a**, H_2_SO_4_/H_2_O for **5b**–**5f**.

Following this, the use of arenes **4a**–**4f** allowed obtaining the 3-arylphthalides **5a**–**5g** (Figure 3).

Synthesis starts with the commercial phthalide (**1**) that was subjected to benzylic radical bromination with *N*-bromosuccinimide (NBS), yielding quantitatively 3-bromophthalide (**2**). Compound **2** was treated with aqueous KOH under reflux, yielding 3-hydroxyphthalide (**3**). Then, the treatment of **3** with acidic conditions led to the corresponding electrophilic phthalidyl ion that underwent condensation with arenes **4a**–**4f,** giving rise to the 3-arylphthalide derivatives **5a**–**5f**, respectively. The reaction conditions involved the use of H_2_SO_4_/H_2_O (3:7) mixtures, except for the synthesis of **5a,** which was accomplished with dioxane/H_2_O (1:4, ca. 5% HCl) (Figure 3). The condensation reaction for the synthesis of compounds **5a**–**5f** proceeded smoothly and with good efficiency. Thus, although compound **5a** was previously synthesized from *o*-phthalaldehydic acid with a 75% yield [32], our procedure proved to be more efficient, yielding **5a** quantitatively. Compounds **5b**, **5c**, and **5d** were also efficiently prepared, with yields of 87%, 96%, and 90%, respectively, while compounds **5e** and **5f** were obtained in lower yields (60% and 50%, respectively). These results also demonstrated excellent functional group compatibility.

**Figure 4:**
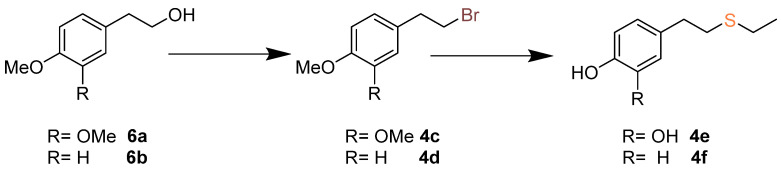
Synthesis of arene derivatives **4c**–**4f**.

Thus, the condensation of **3** with the commercially available compounds resorcinol (**4a**) and guaiacol (**4b**) yielded phthalides **5a** and **5b,** respectively. For the synthesis of compounds **5c**–**5f,** it was necessary to prepare the arenes **4c**–**4f**, respectively (Figure 4). Thus, compounds **4c** and **4d** were obtained from the commercial compounds **6a** and **6b** by treatment with PPh_3_ and CBr_4_ under Apple conditions, causing the displacement of the hydroxy group and the installation of a halogen on the side chain. Demethylation of compounds **4c** and **4d** with EtSNa led to the corresponding hydroxy groups along with the concomitant nucleophilic substitution of the bromine atom by the ethylthio group on the alkyl side chain. On the other hand, the phthalide **5g** was synthesized by the treatment of **5d** with EtSNa (Figure 3). The structures of all target compounds were characterized by extensive spectroscopic analyses (NMR and IR) and HRMS-ESI.

The synthesized phthalides **5a**–**5g** contain a stereogenic center at C-3, and due to the lack of stereochemical control in the synthetic methodology used, these compounds were obtained as racemic mixtures. Biological studies described for various 3-substituted phthalides have shown that the configuration at C-3 does not seem to be determinant for bioactivity [33]. Nonetheless, we were encouraged to evaluate, if possible, phthalides **5a**–**5g** as pure enantiomers. We attempted to obtain optically pure enantiomers of **5a** by chiral HPLC, but none of the chromatographic conditions used led to the separation of the enantiomers. In all cases, low resolution chromatograms were obtained, which suggested an inherent racemization process, likely due to the γ-lactone ring opening favored by the ability of the phenolic ring to form an *o*- or *p*-quinone methide (Figure S15). Considering the pitfall associated with the use of chiral HPLC in 5a, we investigated other alternatives, such as the protection of the hydroxy groups by esterification, which could preclude the formation of quinone methide structures and thus racemization at C-3. In fact, a racemic mixture of esters of **5a** was successfully separated by chiral HPLC into two enantiomers. However, when each enantiomerically pure ester was subjected to hydrolysis to remove the ester group, only the racemic compound **5a** was recovered. These results again suggested a rapid racemization of pure enantiomers of **5a**. On the other hand, we also subjected compound **5f** to chiral HPLC, which in this case led to the corresponding pure enantiomers. However, the value of the specific optical rotations of these enantiomers [α](ca. +10.0° and −9.5°) were lower than those of structurally similar compounds described in reference [34]. Moreover, we noticed that the optical rotation decreased with time until turning null in a one-hour time period. Similar results were also obtained with **5e**.

Interestingly, most natural 3-arylphthalides which bear hydroxy groups in the aryl ring at C-3 have been described as racemates [24] or with unknown stereochemistry at C-3 [35,36], suggesting the racemization of these compounds. Since enzymes act in stereospecific routes leading to pure enantiomers, it is plausible to conclude that the racemization observed in some natural 3-arylphthalides is produced in a post-biosynthetic process. It is also worth noting that although several asymmetric syntheses of 3-arylphthalides can be found in literature [34,37,38,39,40,41,42,43,44], none of them include in their scope the synthesis of compounds bearing phenolic rings at C-3. The easy racemization of 3-arylphthalides which bear *orto*- or *para*-hydroxy groups in the aryl ring at C-3 has been explained in terms of the formation at C-3 of a cationic intermediate stabilized by the ability to form quinone methide structures under almost neutral conditions [45].

Once established that racemization in solution precluded the evaluation of the biological activity of the pure enantiomers of **5a**, **5e**, and **5f**, we decided to perform the activity assays of all synthesized compounds **5a**–**5g** as racemates.

### 2.2. Pharmacology

#### 2.2.1. Antioxidant Activity

The in vitro antioxidant properties of compounds **5a**–**5g** were assessed by using the ABTS assay and Trolox as positive control. Compounds **5c**, **5d**, and **5g** were inactive while compounds **5a**, **5b**, **5e**, and **5f** displayed significant activities. Compound **5a** exhibited slightly better activity than the Trolox standard, while the activity values of **5b**, **5e**, and **5f** were 57%, 43%, and 56% that of the Trolox, respectively (Table 1).

As expected, only compounds bearing hydroxy groups on the aromatic ring at C-3 (**5a**, **5b**, **5e**, **5f**) displayed significant antioxidant activity. Moreover, the results showed that the number of hydroxy groups and their relative position on the benzene ring are crucial for the activity. The antioxidant properties of compounds exhibiting free phenolic groups is well-known, and in particular, the radical-scavenging activity of the natural 3-arylphthalide isopestacin was already reported [24]. Herein, we have shown the activity of the related analogue **5a**. On the other hand, it has been described that the introduction of methoxy groups can increase the antioxidant activity in simple phenolic compounds and in compounds having conjugated systems such as stilbenes and flavonoids [25]. Compound **5b** exhibited higher antioxidant activity than **5e**, which presents the same relative position of the oxygenated substituents but lacks a methoxy group. Although sulfur-containing compounds have been described to reduce oxidative stress, our results are not conclusive [26].

#### 2.2.2. Anti-Inflammatory Activity

The anti-inflammatory activity of the synthesized compounds **5a**–**5g** was tested in assays aimed to detect the inhibition of nitric oxide (NO) production in both immune cell line Bv.2 (microglia) and RAW 264.7 (macrophages) cells. For the more active compounds, the expression of the pro-inflammatory cytokines *Tnfa*, *Il1b*, and *Il6* was also tested.

First, to ensure the safety of the compounds and obtain reliable results in the anti-inflammatory evaluation, the cytotoxicity of the compounds on Bv.2 and RAW 264.7 cells was checked (Figures S16 and S17). We found that compounds **5a**–**5g** exhibited no cytotoxicity at concentrations equal to or below 10 μM. This concentration was selected to evaluate their anti-inflammatory activity.

For the anti-inflammatory assays, Bv.2 and RAW 264.7 cells were stimulated with lipopolysaccharide (LPS) to trigger the release of inflammatory mediators such as NO and pro-inflammatory cytokines, which cause local inflammation upon binding to membrane receptors of cells.

**Figure 5:**
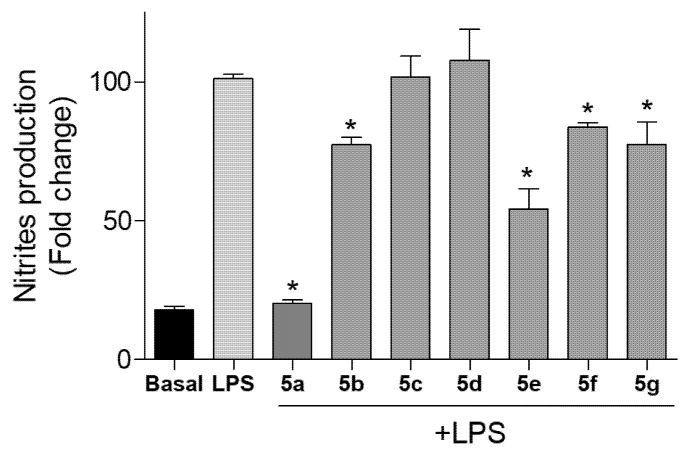
Effects of compounds **5a**–**5g** on NO release in microglial cells. Bv.2 microglial cells were cotreated with the compound at 10 µM and 200 ng/mL LPS for 24 h. Nitrite accumulation in the culture media was measured using the Griess reagent. Results were expressed as a fold change relative to the LPS condition and mean ± SD (n ≥ 3 independent experiments performed in duplicate). Significant differences were determined by one-way ANOVA followed by Bonferroni *t* test; * *p* ≤ 0.05 vs. LPS.

**Figure 6:**
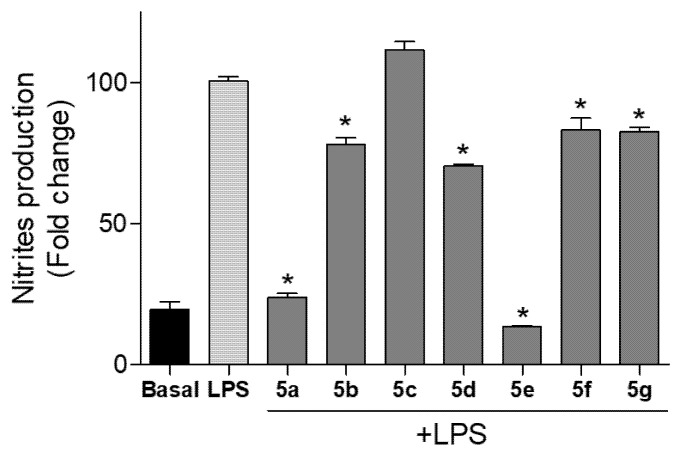
Effects of compounds **5a**–**5g** on NO release in macrophage cells. RAW 264.7 cells were cotreated with the compound at 10 µM and 200 ng/mL LPS for 24 h. Nitrite accumulation in the culture media was measured using the Griess reagent. Results were expressed as a fold change relative to the LPS condition and mean ± SD (n ≥ 3 independent experiments performed in duplicate). Significant differences were determined by one-way ANOVA followed by Bonferroni *t* test; * *p* ≤ 0.05 vs. LPS.

In this study, cells were cotreated with compounds **5a**–**5g** and LPS. The concentration of nitrite (NO_2_^−^), which is one of the major metabolites derived from NO, was measured. The level of nitrites did not change in Bv.2 and RAW 264.7 cells treated with the compounds at 10 μM (data not shown). However, the level of NO production was significantly increased in cells stimulated with LPS. When these cells were cotreated with LPS and compounds **5a**–**5g** the production of nitrites was significantly inhibited (Figure 5 and Figure 6).

In the case of Bv.2 cells (Figure 5), the most active compounds were **5a** and **5e**, causing 79.84% and 45.82% inhibition, respectively, in nitrite production with respect to LPS-stimulated but non-treated cells. Compounds **5b**, **5f**, and **5g** were less active showing inhibition of NO production below 23%, while **5c** and **5d** showed no inhibition.

Similar results were obtained in assays with RAW 264.7 cells (Figure 6). After the cotreatment with LPS and compounds **5a** and **5e** the production of nitrites was significantly inhibited by 76.31% and 86.47%, respectively, with respect to LPS-stimulated but non-treated cells. Compounds **5b**, **5d**, **5f**, and **5g** again showed less activity with NO inhibition levels below 30%, while compound **5c** did not show NO inhibition.

Although the number of compounds is limited, the results strongly suggest that the presence of two hydroxy groups on the aromatic ring at C-3 of the phthalide correlates with anti-inflammatory activity, while the presence of lateral side-chains with either bromine or sulfur seems to lack of significance for the inhibitory activity. Thus, compounds **5a** and **5e**, with two OH substituents on the C-3 aryl ring, caused significant inhibition of LPS-induced NO production in both types of cells. There were significant differences in the effects caused by **5a** (*o*- and *p*-OH) and **5e** (*m*- and *p*-OH) on Bv.2 cells, suggesting that the position of the hydroxy groups is a limiting factor for determining the NO inhibition. However, this trend was not maintained in RAW 264.7 cells, where compounds **5a** and **5e** showed similar levels of NO inhibition. Compounds **5b** and **5f**, which only have one hydroxy group, were less active than **5a** and **5e**, and the position of the hydroxy group (*p*-OH for **5b** and *o*-OH for **5f**) seems irrelevant. Compounds **5c** and **5d** lacking of hydroxy groups did not show apparent NO inhibitory activity in Bv.2 and RAW 264.7 cells, while **5g** only showed weak NO inhibitory activity.

Our study describes for the first time the anti-inflammatory activity of 3-arylphthalides. Previous studies have reported the capability of (Z)-ligustilide (Figure 1) [14,16], the 3-alkylphthalide cnidilide [19] and a few related compounds [20,21,23] to inhibit LPS-induced NO production in RAW 264.7 macrophages. However, most of these compounds only at concentrations higher than 50 μM caused inhibitions higher than 50%, and for ligustilide IC_50_ values of 32.3 μM [14] or 8.45 μM [16] have been reported. These activity levels are significantly lower than those found herein for compound **5a**.

**Figure 7:**
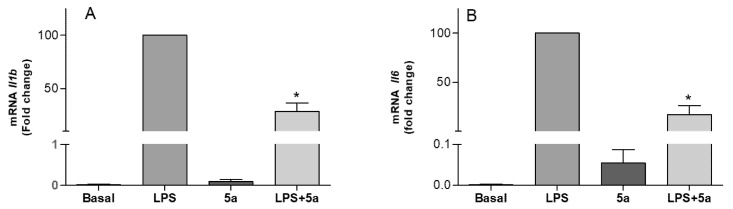
Inhibitory effect of compound **5a** on mRNA pro-inflammatory cytokines expression. (**A**) *Il1b* and *Actin-b* mRNA levels in RAW 264.7 macrophage cells were determined by qRT-PCR and (**B**) *Il6* and *Actin-b* mRNA levels in RAW 264.7 macrophage cells were determined by qRT-PCR. Results were expressed as a fold change relative to the LPS condition and mean ± SD (n ≥ 3 independent experiments performed in duplicate). Significant differences were determined by one-way ANOVA followed by Bonferroni *t*-test; * *p* ≤ 0.05 vs. LPS.

According to these results, compound **5a** combined the highest antioxidant activity in the ABTS assay and the highest potency on the inhibition of NO production. Therefore, compound **5a** was selected to evaluate its effects in the inhibition of the pro-inflammatory cytokine expression in RAW 264.7 cells. LPS-induced inflammatory response is characterized by releasing of pro-inflammatory cytokines such as TNF-α IL-1β, and IL-6. These cytokines play crucial roles during inflammation and are recognized as important early inflammatory mediators, and their over-expression can lead to strong inflammatory reactions. Quantitative RT-PCR analysis revealed a great increase in the mRNA expression of *Il1b* and *Il6* in RAW 264.7 cells after LPS stimulus (Figure 7). However, when cells were cotreated with compound **5a** at 10 μM, the expression of *Il1b* and *Il6* decreased by 71.38% and 82.64%, respectively, while no effect was observed on *Tnfa*. The different behavior of compound **5a** in the downregulation of the cytokines opens the way to forthcoming studies on the mechanism of action by which compound **5a** exerts its anti-inflammatory activity.

These data suggest that compound **5a** could have therapeutic benefit in a variety of pathological conditions in which there is an excessive increase in ROS and pro-inflammatory cytokines. Small molecules such as **5a** do not block the normal actions of cytokines, they only reduce their expression and are less likely to cause the adverse immune effects of the cytokine antagonists [46,47].

## 3. Materials and Methods

### 3.1. General Experimental Procedures

All non-aqueous reactions were performed under an inert atmosphere using flame dried glassware and standard syringe/septa techniques. Tetrahydrofuran (THF) and diethylether (Et_2_O) used for reactions were dried on a SPS Pure Solv system. All other solvents were of HPLC grade and reagents were purchased and used without further purification. Reactions were magnetically stirred and monitored by TLC performed on Merck TLC aluminum sheets (silica gel 60 F_254_). Spots were visualized with UV light (λ = 254 nm) or by staining with cerium sulfate/H_2_SO_4_. Column chromatography was carried out on Merck Silica gel 60 (70–230 mesh) (Merck, Darmstadt, Germany). Chiral HPLC separations were performed on a LaChrom-Hitachi apparatus using a differential refractometer RI-71 or a UV detector L-7400 (Merck, Darmstadt, Germany) working at 254 nm on an OD-H column (Daicel Chiralpak, 4.6 × 250 mm, 5 μm, n-hexane/*i*-PrOH = 80/20 or 90/10, 1.0 mL/min). ABTS (2,2′-azinobis(3-ethylbenzothiazoline-6-sulphonic acid)) diammonium salt and Trolox (6-hydroxy-2,5,7,8-tetramethylchroman-2-carboxylic acid) were purchased from Sigma (St. Louis, MO, USA).

Optical rotations were measured on a Jasco P-2000 polarimeter (Jasco, Easton, MD, USA), operating at the sodium D line with a 100 mm path length cell. Infrared spectra (IR) were recorded on a Perkin-Elmer FT/IR spectrometer (Perkin Elmer, Boston, MA, USA). Frequencies are given as wavenumbers in cm^–1^. ^1^H and ^13^C NMR spectra were recorded on Agilent 400 or Agilent 500 spectrometers (Agilent Technologies, Santa Clara, CA, USA) or Bruker Avance NEO 400 (Bruker BioSpin GmbH, Rheinstetten, Germany) using CD_3_OD, CDCl_3,_ or CD_3_COCD_3_ as solvents. Chemical shifts were referenced using the corresponding solvent signals (δ_H_ 3.30 and δ_C_ 49.0 for CD_3_OD; δ_H_ 7.26 and δ_C_ 77.0 for CDCl_3_; δ_H_ 2.04 and δ_C_ 29.9 for CD_3_COCD_3_). COSY, HSQC, HMBC, and NOESY experiments were performed using standard Agilent pulse sequences. High resolution mass spectra (HRMS) were obtained on a Waters XEVO G2-S Mass spectrometer (Waters, Milford, MA, USA).

### 3.2. Synthetic Procedures

#### 3.2.1. Synthesis of 3-Bromophthalide (**2**)

In a 50 mL, round bottom flask, phthalide (**1**, 1.02 g, 7.57 mmol), NBS (1.52 g, 8.56 mmol) and 51 mg (0.19 mmol) of benzoyl peroxide were dissolved in CCl_4_ (25 mL). The solution was stirred under reflux for 4 h, filtered, and the solvent evaporated under reduced pressure. The reaction mixture was diluted with water (20 mL) and extracted with CH_2_Cl_2_ (3 × 20 mL). The organic layers were combined, dried over anhydrous MgSO_4_, and the solvent taken to dryness yielding 1.60 g of compound **2** (7.52 mmol, quant.).

3-bromophthalide (**2**): ^1^H-NMR (CDCl_3_, 399.945 MHz): Table S1. ^13^C-NMR (CDCl_3_, 100.576 MHz): Table S1. IR (film) υ_max_ 2980, 1768, 1603, 1467, 1090, 1063, 712, 700, 690 cm^−1^. HRESIMS *m*/*z* 212.9558 and 214.9533 [M + H]^+^ (calcd. for C_8_H_6_O_2_^79^Br, 212.9551; calcd. for C_8_H_6_O_2_^81^Br, 214.9531).

#### 3.2.2. Synthesis of 3-Hydroxyphthalide (**3**)

A solution of 3-bromophthalide (**2**, 500 mg, 2.35 mmol) in 25 mL of distilled H_2_O was treated with 85% KOH (200 mg, 3.0 mmol) and stirred under reflux for 2 h. Then the reaction was allowed to warm to rt, treated with KHSO_4_ (170 mg), and extracted with AcOEt (3 × 25 mL). The organic layers were combined, dried over anhydrous MgSO_4_ and the solvent concentrated under reduced pressure, yielding 409 mg of a yellow oil. This oil was purified by column chromatography (SiO_2_, 1.5 × 17 cm, hexanes/AcOEt (6:4)) to afford 304 mg of compound **3** (2.02 mmol, 86%).

3-hydroxyphthalide (**3**): ^1^H-NMR (CD_3_COCD_3_, 399.945 MHz): Table S1. ^13^C-NMR (CD_3_COCD_3_, 100.576 MHz): Table S1. IR (film) υ_max_ 3306, 1761, 1616, 1467, 747, 712, 690 cm^−1^. HRESIMS *m*/*z* 151.0403 [M + H]^+^ (calcd. for C_8_H_7_O_3_, 151.0395).

#### 3.2.3. Synthesis of the Aromatic Derivatives **4c**–**4f**

##### Synthesis of the Bromoderivatives **4c** and **4d**

To a solution of 3.3 mmol of the alcohol 2-(3,4-dimethoxyphenyl)ethanol (**6a**) or 2-(4-methoxyphenyl)ethanol (**6b**) and 1.17 g of PPh_3_ (4.46 mmol) in 20 mL of CH_2_Cl_2_ at 0 °C, and 1.22 g of CBr_4_ (3.63 mmol) were added. The resulting mixture was stirred at rt for 2 h and then concentrated under reduced pressure to give a residue that was purified by column chromatography (SiO_2_, 2 × 17 cm, hexanes/AcOEt 7:3) to yield the corresponding bromoderivatives **4c** and **4d** in quantitative yield.

1,2-dimetoxy-4-(2-bromoethyl)benzene (**4c**): ^1^H-NMR (CDCl_3_, 399.945 MHz): Table S1. ^13^C-NMR (CDCl_3_, 100.576 MHz): Table S1. IR (film) υ_max_ 3055, 2939, 1597, 1460, 1206, 1150, 820, 705 cm^−1^. HRESIMS *m*/*z* 245.0175 [M + H]^+^ (calcd. for C_10_H_14_O_2_^79^Br, 245.0177).

1-(2-bromoethyl)-4-methoxybenzene (**4d**): ^1^H-NMR (CD_3_OD, 399.945 MHz): Table S1. ^13^C-NMR (CD_3_OD, 100.576 MHz): Table S1. IR (film) υ_max_ 3060, 2937, 1595, 1460, 1204, 1150, 820, 700 cm^−1^. HRESIMS *m*/*z* 215.0078 [M + H]^+^ (calcd. for C_9_H_12_O^79^Br, 215.0072).

##### Synthesis of **4e** and **4f**

A solution of bromoderivative **4c** or **4d** (0.24 mmol) in 1.5 mL of DMF was added to a round bottom flask containing 207 mg of NaEtS (2.4 mmol) under inert atmosphere. After heating under reflux for 5 h, the reaction was cooled to 0 °C and added to a solution of HCl 5%. Then, the mixture was treated with AcOEt (2 × 5 mL). The organic layers combined, dried over anhydrous MgSO_4_, and the solvent concentrated under reduced pressure, yielding a residue that was purified by column chromatography (SiO_2_, 2 × 16 cm, hexanes/AcOEt (7:3) for compound **4e** and hexanes/AcOEt (9:1) for compound **4f**), to yield 80–90% of the corresponding demethylated compounds.

4-(2-(ethylthio)ethyl)benzene-1,2-diol (**4e**): ^1^H-NMR (CDCl_3_, 399.945 MHz): Table S1.^13^C-NMR (CDCl_3_, 100.576 MHz): Table S1. IR (film) υ_max_ 3330, 2970, 1610, 1523, 1460, 1210, 1071, 720 cm^−1^. HRESIMS *m*/*z* 197.0636 [M − H]^−^ (calcd. for C_10_H_13_O_2_S, 197.0636).

4-(2-(ethylthio)ethyl)phenol (**4f**): ^1^H-NMR (CDCl_3_, 399.945 MHz): Table S1. ^13^C-NMR (CDCl_3_, 100.576 MHz): Table S1. IR (film) υ_max_ 3350, 2970, 1600, 1520, 1465, 1210, 1068, 720 cm^−1^. HRESIMS *m*/*z* 181.0683 [M − H]^−^ (calcd. for C_10_H_13_OS, 181.0687).

#### 3.2.4. Synthesis of Compounds **5a**–**5g**

##### Synthesis of 3-(2,4-dihydroxyphenyl)phthalide (**5a**)

3-Hydroxyphthalide (**3**, 100 mg, 0.67 mmol) was dissolved in 4 mL of a mixture of H_2_O/dioxane (4:1) and the solution was treated with 250 μL of HCl 37% and stirred for 5′. Then, resorcinol (**4a**, 110 mg, 1 mmol) was added and the reaction was stirred at rt until disappearance of the 3-hydroxyphthalide (**3**). After neutralization with NaHCO_3_ (500 mg), the solution was extracted with AcOEt (3 × 15 mL). The organic layers were combined, dried over anhydrous MgSO_4_, and the solvent concentrated under reduced pressure. Purification of the reaction crude by column chromatography (SiO_2_, 2 × 16 cm, hexanes/AcOEt (60:40)) yielded 162 mg of compound **5a** (0.67mmol, 100%).

3-(2,4-dihydroxyphenyl)phthalide (**5a**): ^1^H-NMR (CD_3_COCD_3_, 399.945 MHz): δ 7.85 (1H, d, 7.8 Hz, H-7), δ 7.71 (1H, ddd, 7.4, 7.4, 1.2 Hz, H-5), δ 7.58 (1H, dd, 7.4, 7.4 Hz, H-6), δ 7.48 (1H, d, 7.8 Hz, H-4), δ 6.77 (1H, s, H-3), δ 6.76 (1H, d, 8.4 Hz, H-6′), δ 6.48 (1H, d, 2.4 Hz, H-3′), δ 6.31 (1H, dd, 8.4, 2.4 Hz, H-5′). ^13^C-NMR (CD_3_COCD_3_, 100.576 MHz): δ 171.1 (s, C-1), δ 160.2 (s, C-4′), δ 157.7 (s, C-2′), δ 151.8 (s, C-3a), δ 134.9 (d, C-5), δ 129.8 (d, C-6′), δ 129.7 (d, C-6), δ 127.2 (s, C-7a), δ 125.6 (d, C-7), δ 124.0 (d, C-4), δ 115.5 (s, C-1′), δ 108.0 (d, C-5′), δ 103.8 (d, C-3′), δ 79.3 (d, C-3). IR (film) υ_max_ 3320, 2970, 1740, 1615, 1520, 1465, 1210, 1170, 1070, 740 cm^−1^. HRESIMS *m*/*z* 243.0675 [M + H]^+^ (calcd. for C_14_H_11_O_4_, 243.0657).

##### Synthesis of Compounds **5b**–**5f**

3-hydroxyphthalide (**3**, 95 mg, 0.63 mmol) was treated with a mixture H_2_SO_4_/H_2_O (3:7) and stirred for 10′. Then, the corresponding aromatic derivative (**4b**–**4f**, 1 mmol) was added and the reaction was stirred at rt until disappearance of the starting material. The reaction was neutralized with NaOH and extracted with CHCl_3_ (3 × 20 mL). The organic layers were combined, dried over anhydrous MgSO_4_, and the solvent concentrated under reduced pressure.

The crude of the reaction between **3** and **4b** was purified by column chromatography (SiO_2_, 2 × 16 cm, hexanes/AcOEt (80:20)), yielding 102 mg of compound **5b** (0.55 mmol, 87%).

The crude of the reaction between **3** and **4c** was purified by column chromatography (SiO_2_, 2 × 17 cm, hexanes/AcOEt (70:30)), yielding 227 mg of compound **5c** (0.60 mmol, 96%).

The crude of the reaction between **3** and **4d** was purified by column chromatography (SiO_2_, 2 × 17 cm, hexanes/AcOEt (70:30)), yielding 198 mg of compound **5d** (0.57 mmol, 90%).

The crude of the reaction between **3** and **4e** was purified by column chromatography (SiO_2_, 2 × 17 cm, hexanes/AcOEt (60:40)), yielding 125 mg of compound **5e** (0.40 mmol, 60%).

The crude of the reaction between **3** and **4f** was purified by column chromatography (SiO_2_, 2 × 17 cm, hexanes/AcOEt (85:15)), yielding 98 mg of compound **5f** (0.31 mmol, 50%).

3-(4-hydroxy-3-methoxyphenyl)phthalide (**5b**): ^1^H-NMR (CDCl_3_, 399.945 MHz): δ 7.96 (1H, d, 7.8 Hz, H-7), δ 7.66 (1H, ddd, 7.4, 7.4, 1.2 Hz, H-5), δ 7.56 (1H, dd, 7.4, 7.4 Hz, H-6), δ 7.33 (1H, d, 7.8 Hz, H-4), δ 6.91 (1H, d, 8.2 Hz, H-5′), δ 6.83 (1H, dd, 8.2, 2.0 Hz, H-6′), δ 6.65 (1H, d, 2.0, H-2′), δ 6.35 (1H, s, H-3), δ 3.82 (3H, s, -OMe). ^13^C-NMR (CDCl_3_, 100.576 MHz): δ 170.5 (s, C-1), δ 149.7 (s, C-3a), δ 146.9 (s, C4′), δ 146.6 (s, C-3′), δ 134.2 (d, C-5), δ 129.3 (d, C-6), δ 128.0 (s, C-1′), δ 125.8 (s, C-7a), δ 125.6 (d, C-7), δ 122.9 (d, C-4), δ 120.8 (d, C-6′), δ 114.5 (d, C-5′), δ 109.3 (d, C-2′), δ 83.0 (d, C-3), δ 56.0 (q, -OMe). IR (film) υ_max_ 3410, 3030, 2935, 2843, 1758, 1610, 1515, 1465, 1280, 1065, 720 cm^−1^. HRESIMS *m*/*z* 257.0819 [M + H]^+^ (calcd. for C_15_H_13_O_4_, 257.0814).

3-(2-(2-bromoethyl)-4,5-dimethoxyphenyl)phthalide (**5c**): ^1^H-NMR (CDCl_3_, 399.945 MHz): δ 7.99 (1H, brd, 7.8 Hz, H-7), δ 7.70 (1H, ddd, 7.8, 7.4, 1.2 Hz, H-5), δ 7.61 (1H, dd, 7.8, 7.4 Hz, H-6), δ 7.34 (1H, dd, 7.8, 0.8 Hz, H-4), δ 6.79 (1H, s, H-3′), δ 6.68 (1H, s, H-3), δ 6.24 (1H, s, H-6′), δ 3.90 (3H, s, -OMe), δ 3.75-3.55 (2H, m, -CH_2_-CH_2_-Br), δ 3.62 (3H, s, -OMe), δ 3.35 (2H, m, -CH_2_-CH_2_-Br). ^13^C-NMR (CDCl_3_, 100.576 MHz): δ 170.3 (s, C-1), δ 150.0 (s, C-4′), δ 149.2 (s, C-3a), δ 148.3 (s, C-5′), δ 134.3 (d, C-5), δ 131.4 (s, C-2′), δ 129.5 (d, C-6), δ 126.5 (s, C-7a), δ 126.0 (s, C-1′), δ 125.8 (d, C-7), δ 123.1 (d, C-4), δ 113.1 (d, C-3′), δ 110.6 (d, C-6′), δ 79.6 (d, C-3), δ 56.0 (q, -OMe), δ 55.9 (q, -OMe), δ 35.8 (t, -CH_2_-CH_2_-Br), δ 32.7 (t, -CH_2_-CH_2_-Br). IR (film) υ_max_ 3000, 2940, 2875, 1760, 1590, 1465, 1200, 1056, 1000, 830, 690 cm^−1^. HRESIMS *m*/*z* 377.0389 [M + H]^+^ (calcd. for C_18_H_18_O_4_^79^Br, 377.0388).

3-(5-(2-bromoethyl)-2-methoxyphenyl)phthalide (**5d**): ^1^H-NMR (CDCl_3_, 399.945 MHz): δ 7.94 (1H, d, 7.8 Hz, H-7), δ 7.62 (1H, ddd, 7.4, 7.4, 1.2 Hz, H-5), δ 7.52 (1H, dd, 7.4, 7.4 Hz, H-6), δ 7.46 (1H, dd, 7.8, 0.8 Hz, H-4), δ 7.17 (1H, dd, 8.2, 1.9, H-4′), δ 6.93 (1H, d, 8.2 Hz, H-3′), δ 6.92 (1H, d, 1.9 Hz, H-6′), δ 6.84 (1H, s, H-3), δ 3.91 (3H, s, -OMe), δ 3.44 (2H, brdd, 7.8, 7.4 Hz, -CH_2_-Br), δ 3.02 (2H, dd, 7.8, 7.4 Hz, -CH_2_-CH_2_-Br). ^13^C-NMR (CDCl_3_, 100.576 MHz): δ 171.0 (s, C-1), δ 155.9 (s, C-2′), δ 150.4 (s, C-3a), δ 134.2 (d, C-5), δ 131.4 (s, C-5′), δ 130.2 (d, C-4′), δ 129.1 (d, C-6), δ 126.9 (d, C-6′), δ 125.5 (d, C-7), δ 125.3 (2 × s, C-1′ and C-7a), δ 122.9 (d, C-4), δ 111.1 (d, C-3′), δ 77.9 (d, C-3), δ 55.7 (q, -OMe), δ 38.4 (t, -CH_2_-CH_2_-Br), δ 33.0 (t, -CH_2_-CH_2_-Br). IR (film) υ_max_ 3030, 2935, 2836, 1763, 1612, 1460, 1170, 1045, 720, 690 cm^−1^. HRESIMS *m*/*z* 347.0288 [M + H]^+^ (calcd. for C_17_H_16_O_3_^79^Br, 347.0283).

3-(2-(2-(ethylthio)ethyl)-4,5-dihydroxyphenyl)phthalide (**5e**): ^1^H-NMR (CD_3_OD, 399.945 MHz): δ 7.92 (1H, d, 7.8 Hz, H-7), δ 7.75 (1H, ddd, 7.8, 7.8, 1.0 Hz, H-5), δ 7.63 (1H, dd, 7.8, 7.8 Hz, H-6), δ 7.41 (1H, dd, 7.8, 0.8 Hz, H-4), δ 6.78 (1H, s, H-3), δ 6.74 (1H, s, H-3′), δ 6.15 (1H, s, H-6′), δ 3.05-2.84 (2H, m, -CH_2_-CH_2_-S-), δ 2.84-2.72 (2H, m, -CH_2_-CH_2_-S-), δ 2.54 (2H, q, 7.4 Hz, -S-CH_2_CH_3_), δ 1.23 (3H, t, 7.4 Hz, -S-CH_2_CH_3_). ^13^C-NMR (CD_3_OD, 100.576 MHz): δ 172.8 (s, C-1), δ 151.7 (s, C-3a), δ 147.7 (s, C-4′), δ 145.2 (s, C-5′), δ 135.7 (d, C-5), δ 133.9 (s, C-1′), δ 130.5 (d, C-6), δ 127.6 (s, C-7a), δ 126.1 (d, C-7), δ 126.0 (s, C-2′), δ 124.7 (d, C-4), δ 118.2 (d, C-3′), δ 115.7 (d, C-6′), δ 82.0 (d, C-3), δ 34.6 (t, -CH_2_-CH_2_-S-), δ 33.7 (t, CH_2_-CH_2_-S-), δ 26.9 (t, -S-CH_2_CH_3_), δ 15.2 (q, -S-CH_2_CH_3_). IR (film) υ_max_ 3300, 3025, 2950, 1750, 1600, 1520, 1465, 1260, 1065, 720 cm^−1^. HRESIMS *m*/*z* 329.0847 [M − H]^−^ (calcd. for C_18_H_17_O_4_S, 329.0848).

3-(5-(2-(ethylthio)ethyl)-2-hydroxyphenyl)phthalide (**5f**): ^1^H-NMR (CD_3_OD, 399.945 MHz): δ 7.88 (1H, d, 7.8 Hz, H-7), δ 7.68 (1H, ddd, 7.8, 7.8, 1.2 Hz, H-5), δ 7.56 (1H, dd, 7.4, 7.4 Hz, H-6), δ 7.51 (1H, dd, 7.8, 0.8 Hz, H-4), δ 7.04 (1H, dd, 8.2, 2.3 Hz, H-4′), δ 6.84 (1H, s, H-3), δ 6.80 (1H, brs, H-6′), δ 6.79 (1H, d, 8.2, H-3′), δ 2.72 (2H, m, -CH_2_-CH_2_-S-), δ 2.65 (2H, m, -CH_2_-CH_2_-S-), δ 2.48 (2H, q, 7.4 Hz, -S-CH_2_CH_3_), δ 1.15 (3H, t, 7.4 Hz, -S-CH_2_CH_3_). ^13^C-NMR (CD_3_OD, 100.576 MHz): δ 173.4 (s, C-1), δ 155.1 (s, C-2′), δ 152.5 (s, C-3a), δ 135.6 (d, C-5), δ 133.3 (s, C-5′), δ 131.3 (d, C-4′), δ 130.2 (d, C-6), δ 128.1 (d, C-6′), δ 126.7 (s, C-7a), δ 126.0 (d, C-7), δ 124.2 (d, C-4), δ 124.1 (s, C-1′), δ 116.7 (d, C-3′), δ 80.5 (d, C-3), δ 36.5 (t, CH_2_-CH_2_-S-), δ 34.3 (t, -CH_2_-CH_2_-S-), δ 26.7 (t, -S-CH_2_CH_3_), δ 15.1 (q, -S-CH_2_CH_3_). IR (film) υ_max_ 3350, 3028, 2935, 2843, 1760, 1618, 1510, 1465, 1280, 1060, 720 cm^−1^. HRESIMS *m*/*z* 315.1049 [M + H]^+^ (calcd. for C_18_H_19_O_3_S, 315.1055).

##### Synthesis of 3-(5-(2(Ethylthio)ethyl)-2-Methoxyphenyl)phthalide (**5g**)

A solution of compound **5d** (50 mg, 0.14 mmol) in 2.0 mL of DMF was added to a flask containing 118 mg of NaEtS (1.4 mmol) under inert atmosphere. After heating under reflux for 3 h, the reaction was cooled to 0 °C and added to a solution of HCl 5%. Then, the mixture was treated with AcOEt (2 × 10 mL). The combined organic layers were dried over anhydrous MgSO_4_, the solvent concentrated under reduced pressure and the residue was purified by column chromatography (SiO_2_, 1 × 15 cm, hexanes/AcOEt (80:20)), yielding 15 mg (0.045 mmol, 33%) of the compound **5g**.

3-(5-(2(ethylthio)ethyl)-2-methoxyphenyl)phthalide (**5g**): ^1^H-NMR (CDCl_3_, 499.720 MHz): δ 7.93 (1H, brd, 7.8 Hz, H-7), δ 7.61 (1H, ddd, 7.8, 7.5, 1.2 Hz, H-5), δ 7.52 (1H, dd, 7.8, 7.4 Hz, H-6), δ 7.45 (1H, dd, 7.8, 0.8 Hz, H-4), δ 7.15 (1H, dd, 8.4, 2.3 Hz, H-4′), δ 6.92 (1H, d, 2.3 Hz, H-6′), δ 6.90 (1H, d, 8.4 Hz, H-3′), δ 6.83 (1H, s, H-3), δ 3.90 (3H, s, -OMe), δ 2.74 (2H, m, -CH_2_-CH_2_-S-), δ 2.67 (2H, m, -CH_2_-CH_2_-S-), δ 2.50 (2H, q, 7.4 Hz, -S-CH_2_CH_3_), δ 1.21 (3H, t, 7.4 Hz, -S-CH_2_CH_3_). ^13^C-NMR (CDCl_3_, 125.669 MHz): δ 171.0 (s, C-1), δ 155.4 (s, C-2′), δ 150.5 (s, C-3a), δ 134.1 (d, C-5), δ 133.2 (s, C-5′), δ 129.9 (d, C-4′), δ 129.0 (d, C-6), δ 126.7 (d, C-6′), δ 125.5 (s, C-7a), δ 125.5 (d, C-7), δ 125.0 (s, C-1′), δ 123.0 (d, C-4), δ 111.0 (d, C-3′), δ 78.0 (d, C-3), δ 55.7 (q, -OMe), δ 35.3 (t, -CH_2_-CH_2_-S-), δ 33.2 (t, -CH_2_-CH_2_-S-) δ 26.0 (t, -S-CH_2_CH_3_), δ 14.8 (q, -S-CH_2_CH_3_). IR (film) υ_max_ 3028, 2940, 2855, 1760, 1610, 1580, 1454, 1280, 1060, 722, 690 cm^−1^. HRESIMS *m*/*z* 329.1217 [M + H]^+^ (calcd. for C_19_H_21_O_3_S, 329.1211).

### 3.3. Antioxidant ASSAY

Antioxidant activity was determined by the ABTS free-radical decolorization assay developed by Re et al. [48], with slight modifications. In brief, a solution of the radical cation ABTS^+•^ was prepared by mixing (1:1, *v*/*v*) a solution of ABTS diammonium salt (7 mM) and a solution of potassium persulfate (2.45 mM) in H_2_O. The mixture was kept in the dark at room temperature for 12–18 h before use. Then, the solution was diluted with EtOH to an absorbance of 0.70 ± 0.02 at 734 nm. Stock solutions of Trolox (standard) and of the tested compounds were prepared in EtOH. For the assay, 100 μL of the Trolox solution or 100 μL of tested compound solution were mixed with 2 mL of the ABTS^+•^ solution. The final concentrations of Trolox or tested compounds were 1, 5, 10, 20, 25, and 30 µM. Controls were prepared by adding 100 μL of EtOH to 2 mL of ABTS^+•^ solution. Six minutes after mixing, the absorbance at 734 nm was measured after 6 min in a UV-Vis spectrophotometer, VWR UV-1600PC (VWR, Radnor, PA, USA). All determinations were carried out in triplicate. The percentage of inhibition of the absorbance was calculated by the following equation: % Inhibition = [(A0 − A1)/A0] × 100, where A0 expresses the absorbance of control and A1 the absorbance of the tested compound.

### 3.4. Anti-Inflammatory Activity

#### 3.4.1. Cell Culture

Mouse microglia Bv.2 cell line was supplied by Dr. M.L. Nieto (IBGM, Spain). Mouse macrophage RAW 264.7 cell line was supplied by Dr. A.M. Valverde (IIBm “Alberto Sols” UAM-CSIC-Madrid, Spain). An amount of 1.5 × 10^5^ cells/well was seeded in a 6-multiwell plate (Sarstedt, Germany). The culture conditions were 37 °C in a humidified atmosphere with 5% CO_2_ in RPMI supplemented with 10% (*v*/*v*) heat-inactivated fetal bovine serum (FBS), 1% (*v*/*v*) penicillin/streptomycin (Sigma), and 2 mM l-glutamine (Gibco, Carlsbad, CA, USA). All experimental cell approaches were performed in complete medium without FBS.

#### 3.4.2. Analysis of the Cellular Viability by Crystal Violet Staining

Cells were cultured in 24-well plates and grown up to 70% confluence. The cells were treated with solutions of the compounds to reach final concentrations of 0.1, 1.0, 10.0, 25.0, and 50.0 µM and incubated in serum-free medium. After 24 h, the medium was discarded and cells were fixed by adding 0.5 mL of glutaraldehyde 1% (*v*/*v*) for 30 min. Then, the plates were rinsed with phosphate buffer saline (PBS) and the remaining viable adherent cells were stained with crystal violet 0.1% (*w*/*v*) for 30 min. After rinsing plates with water and drying for 24 h, 0.5 mL of acetic acid 10% (*v*/*v*) was added. The absorbance of each plate was read spectrophotometrically at 590 nm in a microplate reader (Power Wave, Bioteck, Torino, Italy).

#### 3.4.3. Analysis of Nitrites (NO_2_^−^)

Cells were cultured in 6-well plates and grown up to 70% confluence. The cells were co-treated with lipopolysaccharide (LPS, 200 ng/mL) and the compounds at 10 µM in serum-free medium for 24 h. Dexamethasone (Dx) was used as positive control at 2.5 µM. At this concentration of Dx, the NO production in LPS-stimulated cells decreased to the basal values. After cell treatments, levels of NO_2_^−^ were measured by using the Griess reagent [49]. Briefly, cell cultured medium was treated with an acid solution containing 1% sulfanilamide and 0.1% *N*-(1-naphthyl)ethylenediamine (NEDA) and read spectrophotometrically at 548 nm in a microplate reader.

#### 3.4.4. Quantitative Real-Time PCR (qPCR) Analysis

Total RNA was extracted with TRI^®^ reagent (Sigma, Madrid, Spain) and reverse-transcribed using the iScript gDNA Clear cDNA Synthesis Kit from BioRad (Madrid, Spain). qPCR was performed with the iTaq Universal Probes Supermix from BioRad (Madrid, Spain) in a CFX Connect Real-Time System from BioRad (Madrid, Spain). Analyses of relative gene expression data were performed using the 2^−∆∆al^ method. Primer–probe sets for mouse *Il1b*, *Il6* and *Tnfa*, and *actin-b* were purchased as predesigned TaqMan probe expression assays (Applied Biosystems, Foster City, CA, USA).

#### 3.4.5. Statistical Analysis

Data are presented as mean ± standard deviation (SD) and were compared by using the ANOVA test and Bonferroni post hoc test. All statistical analyses were performed using GraphPad Prism 8.0 software (GraphPad Software Inc., San Diego, CA, USA). Differences were considered statistically significant at *p* ≤ 0.05.

## 4. Conclusions

In conclusion, the use of a dehydrative coupling reaction between 3-hydroxyphthalide and substituted arenes allowed obtaining a series of 3-arylphthalides with good yields, good levels of site selectivity, and with high functional group tolerance. In antioxidant and anti-inflammatory assays, compounds bearing hydroxy groups on the 3-aryl ring displayed significant activities. In particular, compound **5a** has been identified as possessing strong antioxidant activity and causing high inhibition of LPS-induced NO production in Bv.2 and RAW 264.7 cells. Moreover, compound **5a** significantly decreases mRNA expression of pro-inflammatory cytokines *Il1b* and *Il6* in LPS-stimulated RAW 264.7 cells. These results have disclosed the anti-inflammatory potential of phthalides bearing a phenolic ring at C-3 and provide compelling evidence that slight structural modifications on the aryl ring derivatives conferred a remarkable impact on their antioxidant and anti-inflammatory activities. The reduction of mRNA levels in classical pro-inflammatory cytokines reveals the potential effects on the signaling pathways involved. Thus, further investigation of the inflammasome complex and/or the kinase-stress pathways will be analyzed in order to elucidate the specific anti-inflammatory pathways promoted by the compound **5a** and related 3-arylphthalides.

## 5. Patents

Compounds **5a** and **5e** in this manuscript are the subject of a patent application at the Spanish Patent Office. Application no. P202130739.

## Figures and Tables

**Table 1:** Antioxidant activities of compounds **5a**, **5b**, **5e**, and **5f** in the ABTS assay.

Compound	Trolox	5a	5b	5e	5f
EC_50_ (μM ± SD, n = 3)	9.98 ± 0.09	8.93 ± 0.20	17.37 ± 0.29	23.03 ± 0.23	17.89 ± 0.26

## Data Availability

Data is contained within the article and Supplementary Materials.

## Supplementary Materials

The following supporting information can be downloaded at: https://www.mdpi.com/article/10.3390/ph15050588/s1: Supplementary Table S1, ^1^H and ^13^C NMR data of compounds **2**, **3**, **4c**–**4f**; Supplementary Figures S1–S14, ^1^H and ^13^C NMR spectra of compounds **5a**–**5g**; Supplementary Figure S15, racemization of **5a**; Supplementary Figure S16, cell viability of Bv.2 cells; and Supplementary Figure S17, cell viability of RAW 264.7 cells.
