# Supplementary material for: Synthesis and Antioxidant/Anti-Inflammatory Activity of 3-Arylphthalides
Source: Pharmaceuticals (Basel). 2022 May 10;15(5):588. doi: 10.3390/ph15050588 (PMC9144619; doi:10.3390/ph15050588)
Supplement: Supplementary file 1 [file pharmaceuticals-15-00588-s001.zip › pharmaceuticals-1717180-supplementary.pdf]

## Supplementary material

# Synthesis, Antioxidant, and Anti-inflammatory Activity of 3-Arylphthalides

María J. Ortega [1,*], Belén Parra-Torrejón [1], Fátima Cano-Cano [2,3], Laura Gómez-Jaramillo [2], M. Carmen González-Montelongo and Eva Zubía [1]

[1] Departamento de Química Orgánica, Facultad de Ciencias del Mar y Ambientales, Universidad de Cádiz, 11510 Puerto Real (Cádiz), Spain; belen.parra@uca.es (B.P-T.)

[2] Unidad de Investigación, Instituto de Investigación e Innovación Biomédica de Cádiz (INiBICA), Hospital Universitario Puerta del Mar, Avda. Ana de Viya 21, 11009 Cádiz, Spain; anaarroba@gmail.com (A.I.A.); laugomjar@gmail.com (L.G.-J.); mcmontel@gmail.com (M.C.G.-M.)

*Correspondence: mariajesus.ortega@uca.es

**1. Characterization of compounds 2, 3, 4c-4f**

**Table S1.** $^{1}$H NMR and $^{13}$C NMR data of compounds **2**, **3**, **4c**-**4f**

**2. $^{1}$H NMR and $^{13}$C NMR spectra of compounds 5a-5g**

**Figure S1.** $^{1}$H NMR spectrum (400 MHz, $CD_3COCD_3$) of compound **5a**

**Figure S2.** $^{13}$C NMR spectrum (100 MHz, $CD_3COCD_3$) of compound **5a**

**Figure S3.** $^{1}$H NMR spectrum (400 MHz, $CDCl_3$) of compound **5b**

**Figure S4.** $^{13}$C NMR spectrum (100 MHz, $CDCl_3$) of compound **5b**

**Figure S5.** $^{1}$H NMR spectrum (400 MHz, $CDCl_3$) of compound **5c**

**Figure S6.** $^{13}$C NMR spectrum (100 MHz, $CDCl_3$) of compound **5c**

**Figure S7.** $^{1}$H NMR spectrum (400 MHz, $CDCl_3$) of compound **5d**

**Figure S8.** $^{13}$C NMR spectrum (100 MHz, $CDCl_3$) of compound **5d**

**Figure S9.** $^{1}$H NMR spectrum (400 MHz, $CD_3OD$) of compound **5e**

**Figure S10.** $^{13}$C NMR spectrum (100 MHz, $CD_3OD$) of compound **5e**

**Figure S11.** $^{1}$H NMR spectrum (400 MHz, $CD_3OD$) of compound **5f**

**Figure S12.** $^{13}$C NMR spectrum (100 MHz, $CD_3OD$) of compound **5f**

**Figure S13.** $^{1}$H NMR spectrum (500 MHz, $CDCl_3$) of compound **5g**

**Figure S14.** $^{13}$C NMR spectrum (125 MHz, $CDCl_3$) of compound **5g**

**3. Figure S15.** Proposed mechanism of racemization process in **5a**

**4. Cell viability**

**Figure S16.** Cell survival Bv.2 cells

**Figure S17.** Cell survival RAW 264.7 cells

1. **Characterization of compounds 2, 3, 4c-4f**
   **Table S1**

| Compound | $^1$H NMR and $^{13}$C NMR data |
|---|---|
| 3-bromophthalide (**2**):<br>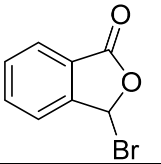 | **$^1$H-NMR ($CDCl_3$, 399.945 MHz):** δ 7.94 (1H, brd, 7.5 Hz, H-7), δ 7.78 (1H, ddd, 7.3, 7.3, 1.1 Hz, H-6), δ 7.64 (1H, brd, 7.5 Hz, H-4), δ 7.62 (1H, dd, 7.6, 7.6 Hz, H-5), δ 7.40 (1H, s, H-3).<br>**$^{13}$C-NMR ($CDCl_3$, 100.576 MHz):** δ 167.3 (s, C-1), δ 148.8 (s, C-3a), δ 135.2 (d, C-5), δ 130.9 (d, C-6), δ 125.9 (d, C-7), δ 124.1 (s, C-7a), δ 123.5 (d, C-4), δ 74.6 (d, C-3). |
| 3-hydroxyphthalide (**3**):<br>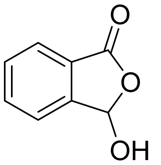 | **$^1$H-NMR ($CD_3COCD_3$, 399.945 MHz):** δ 7.84 (1H, brd, 8.0 Hz, H-7), δ 7.80 (1H, ddd, 7.5, 7.4, 1.0 Hz, H-5), δ 7.72 (1H, brd, 7.7 Hz, H-4), δ 7.66 (1H, brdd, 7.4, 7.4 Hz, H-6), δ 6.75 (1H, s, H-3).<br>**$^{13}$C-NMR ($CD_3COCD_3$, 100.576 MHz):** δ 169.1 (s, C-1), δ 147.9 (s, C-3a), δ 135.0 (d, C-5), δ 131.3 (d, C-6), δ 127.7 (s, C-7a), δ 125.3 (d, C-7), δ 124.3 (d, C-4), δ 98.7 (d, C-3). |
| 1,2-dimetoxy-4-(2-bromoethyl)benzene (**4c**):<br>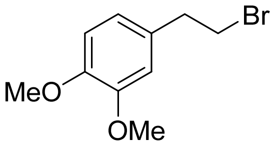 | **$^1$H-NMR ($CDCl_3$, 399.945 MHz):** δ 6.82 (1H, brd, 8.2 Hz, H-6), δ 6.76 (1H, dd, 8.2, 1.9 Hz, H-5), δ 6.72 (1H, brd, 1.9 Hz, H-3), δ 3.88 (3H, s, -OMe), δ 3.86 (3H, s, -OMe), δ 3.54 (2H, t, 7.6 Hz, H-2′), δ 3.10 (2H, t, 7.6 Hz, H-1′).<br>**$^{13}$C-NMR ($CDCl_3$, 100.576 MHz):** δ 148.9 (s, C-2), δ 148.0 (s, C-1), δ 131.5 (s, C-4), δ 120.6 (d, C-5), δ 111.9 (d, C-3), δ 111.3 (d, C-6), δ 55.9 (2 x q,-OMe), δ 39.1 (t, C-1′), δ 33.2 (t, C-2′). |
| 1-(2-bromoethyl)-4-methoxybenzene (**4d**):<br>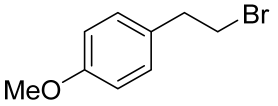 | **$^1$H-NMR ($CD_3OD$, 399.945 MHz):** δ 7.14 (2H, d, 8.6 Hz, H-3, H-5), δ 6.86 (2H, d, 8.6 Hz, H-2, H-6), δ 3.80 (3H, s, -OMe), δ 3.54 (2H, t, 7.6 Hz, H-2′), δ 3.12 (2H, t, 7.6 Hz, H-1′).<br>**$^{13}$C-NMR ($CD_3OD$, 100.576 MHz):** δ 158.5 (s, C-4), δ 131.0 (s, C-1), δ 129.6 (s, C-3, C-5), δ 114.0 (d, C-2, C-6), δ 55.2 (q, -OMe), δ 38.6 (t, C-1′), δ 33.4 (t, C-2′). |
| 4-(2-(ethylthio)ethyl)benzene-1,2-diol (**4e**):<br>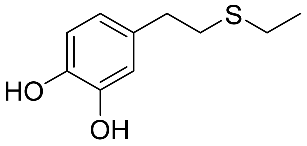 | **$^1$H-NMR ($CDCl_3$, 399.945 MHz):** δ 6.79 (1H, d, 8.4 Hz, H-6), δ 6.73 (1H, d, 1.7 Hz, H-3), δ 6.63 (1H, dd, 8.0, 1.9 Hz, H-5), δ 2.76-2.72 (4H, m, H-1′ y H-2′), δ 2.57 (2H, q, 7.2 Hz, -$C\underline{H_2}CH_3$), δ 1.25 (3H, t, 7.6 Hz, -$CH_2C\underline{H_3}$).<br>**$^{13}$C-NMR ($CDCl_3$, 100.576 MHz):** δ 143.6 (s, C-2), δ 142.0 (s, C-1), δ 133.7 (s, C-4), δ 120.8 (d, C-5), δ 115.6 (d, C-6), δ 115.4 (d, C-3), δ 35.5 (t, C-2′), δ 33.3 (t, C-1′), δ 26.0 (t, -$\underline{C}H_2CH_3$), δ 14.7 (q, -$CH_2\underline{C}H_3$). |
| 4-(2-(ethylthio)ethyl)phenol (**4f**):<br>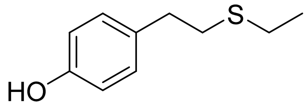 | **$^1$H-NMR ($CDCl_3$, 399.945 MHz):** δ 7.07 (2H, d, 8.8 Hz, H-3, H-5), δ 6.76 (2H, d, 8.8 Hz, H-2, H-6), δ 2.82 (2H, m, H-1′), δ 2.74 (2H, m, H-2′), δ 2.55 (2H, q, 7.6 Hz, -$C\underline{H_2}CH_3$), δ 1.26 (3H, t, 7.6 Hz, -$CH_2C\underline{H_3}$).<br>**$^{13}$C-NMR ($CDCl_3$, 100.576 MHz):** δ 154.0 (s, C-1), δ 132.9 (s, C-4), δ 129.6 (d, C-3, C5), δ 115.3 (d, C-2, C-6), δ 35.4 (t, C-1′), δ 33.4 (t, C-2′), δ 26.1 (t, -$\underline{C}H_2CH_3$), δ 14.7 (q, -$CH_2\underline{C}H_3$). |

## 2. $^{1}H$ NMR and $^{13}C$ NMR spectra of compounds 5a-5g

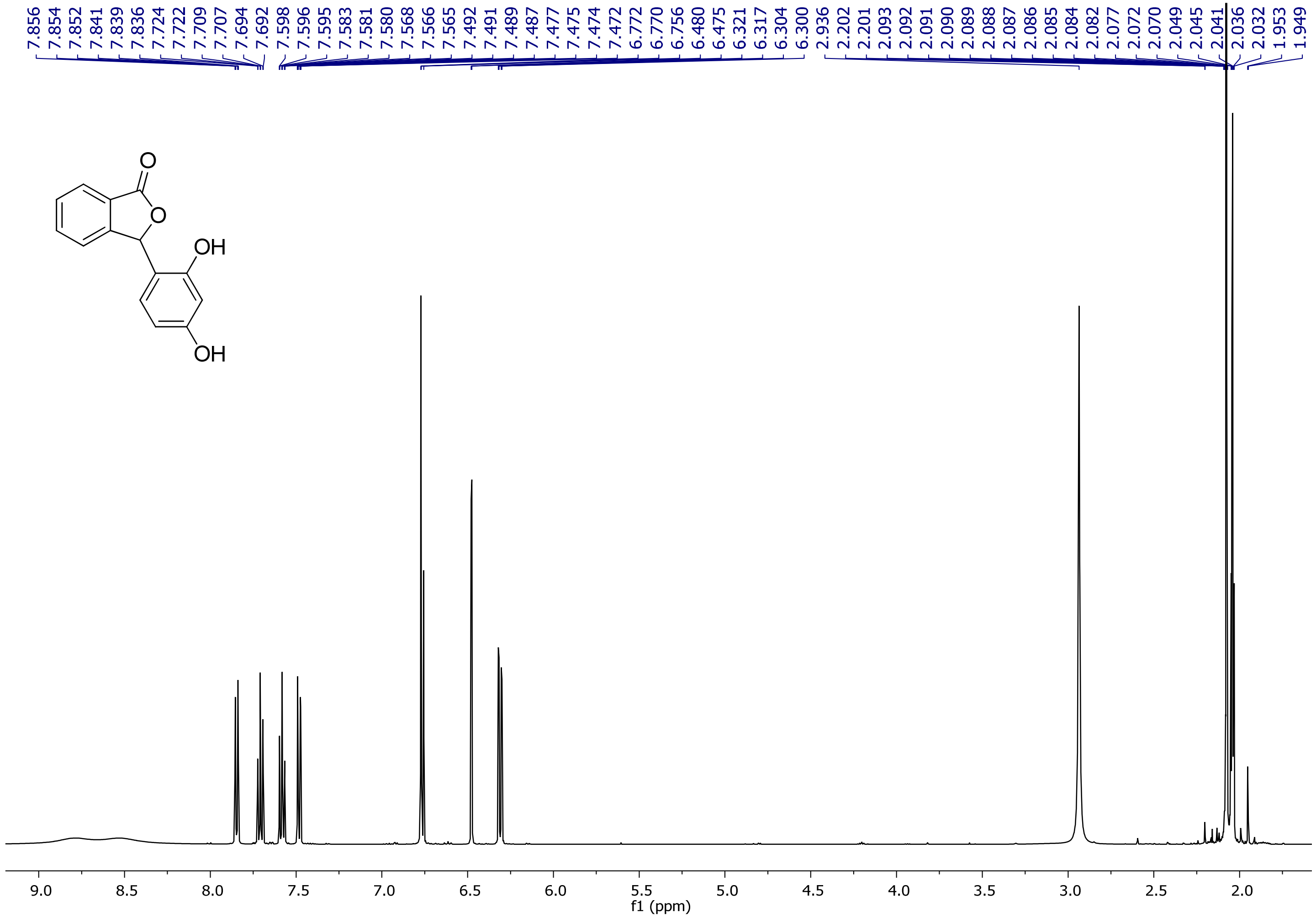


**Figure S1.** $^{1}H$ NMR spectrum (400 MHz, $CD_3COCD_3$) of compound **5a**

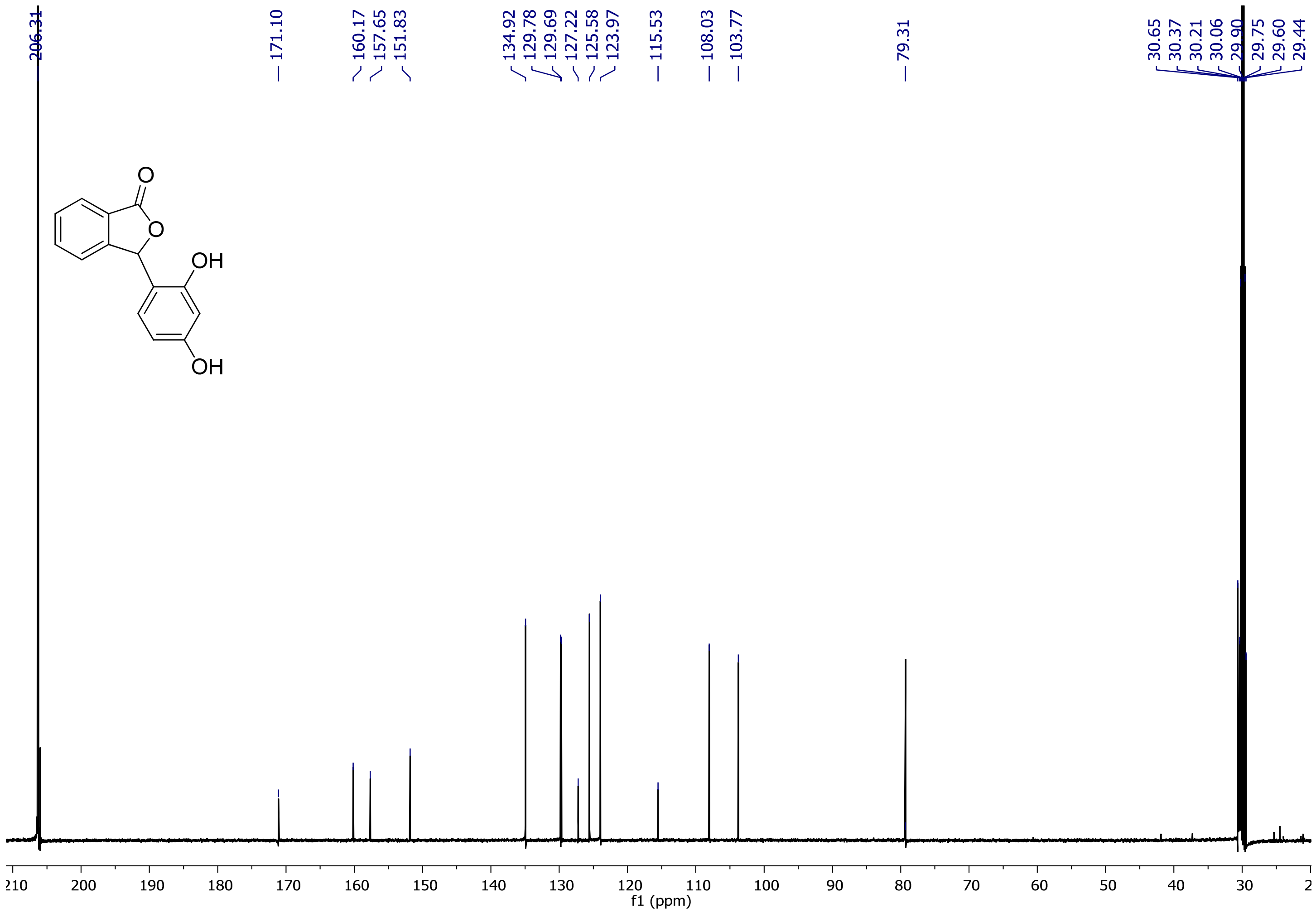


**Figure S2.** $^{13}C$ NMR spectrum (100 MHz, $CD_3COCD_3$) of compound **5a**

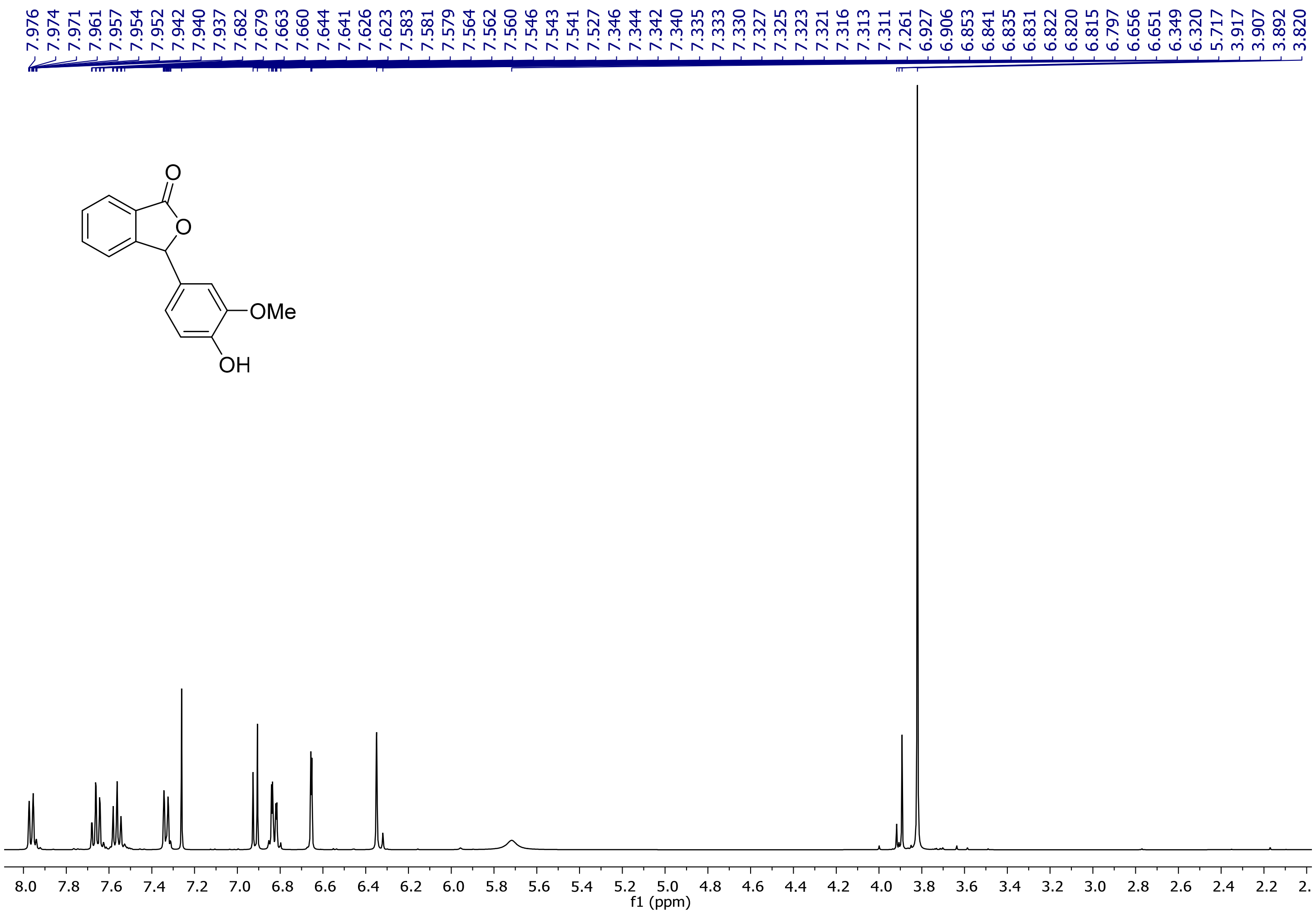


**Figure S3.** $^{1}$H NMR spectrum (400 MHz, $CDCl_3$) of compound **5b**

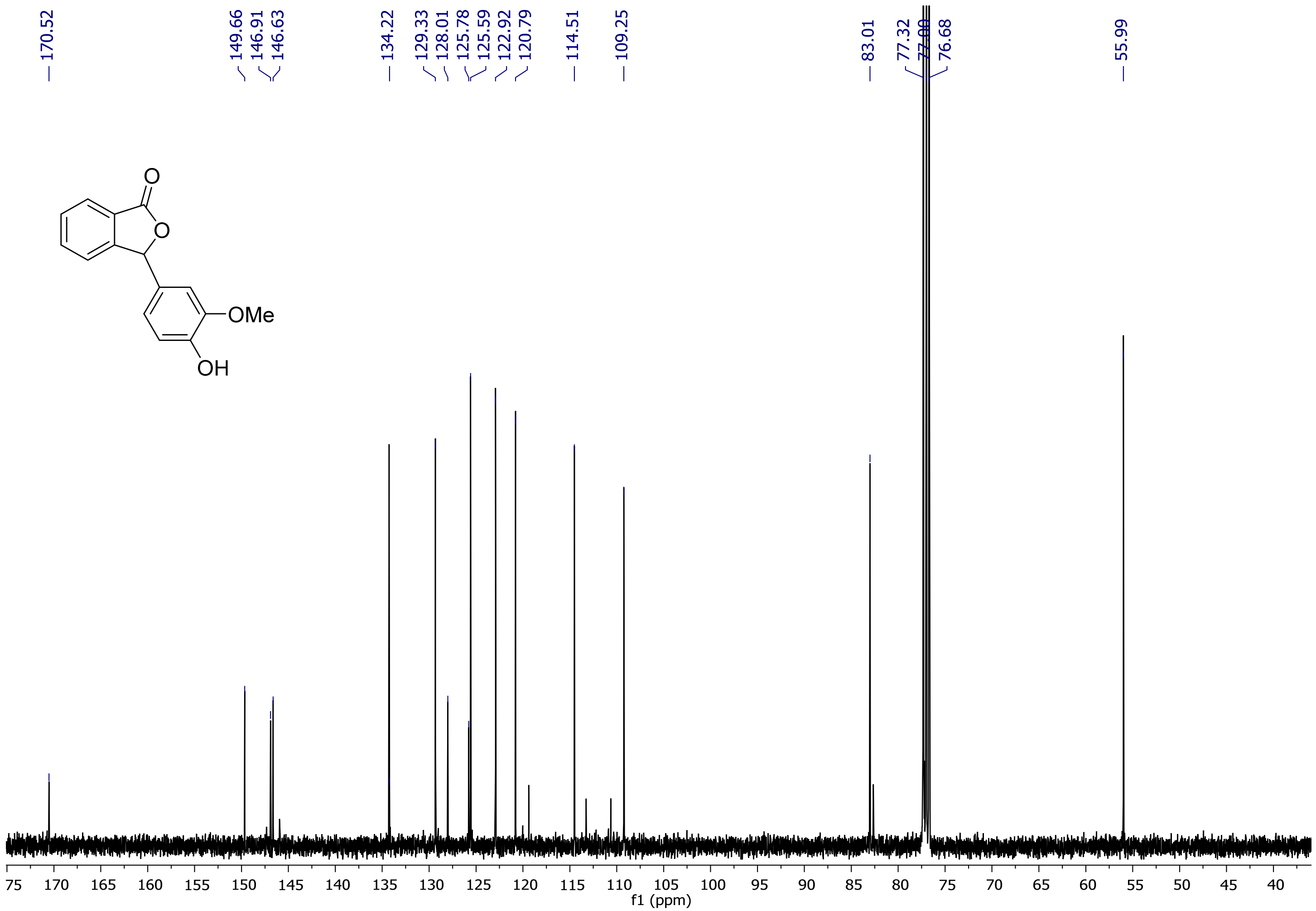


**Figure S4.** $^{13}C$ NMR spectrum (100 MHz, $CDCl_3$) of compound **5b**

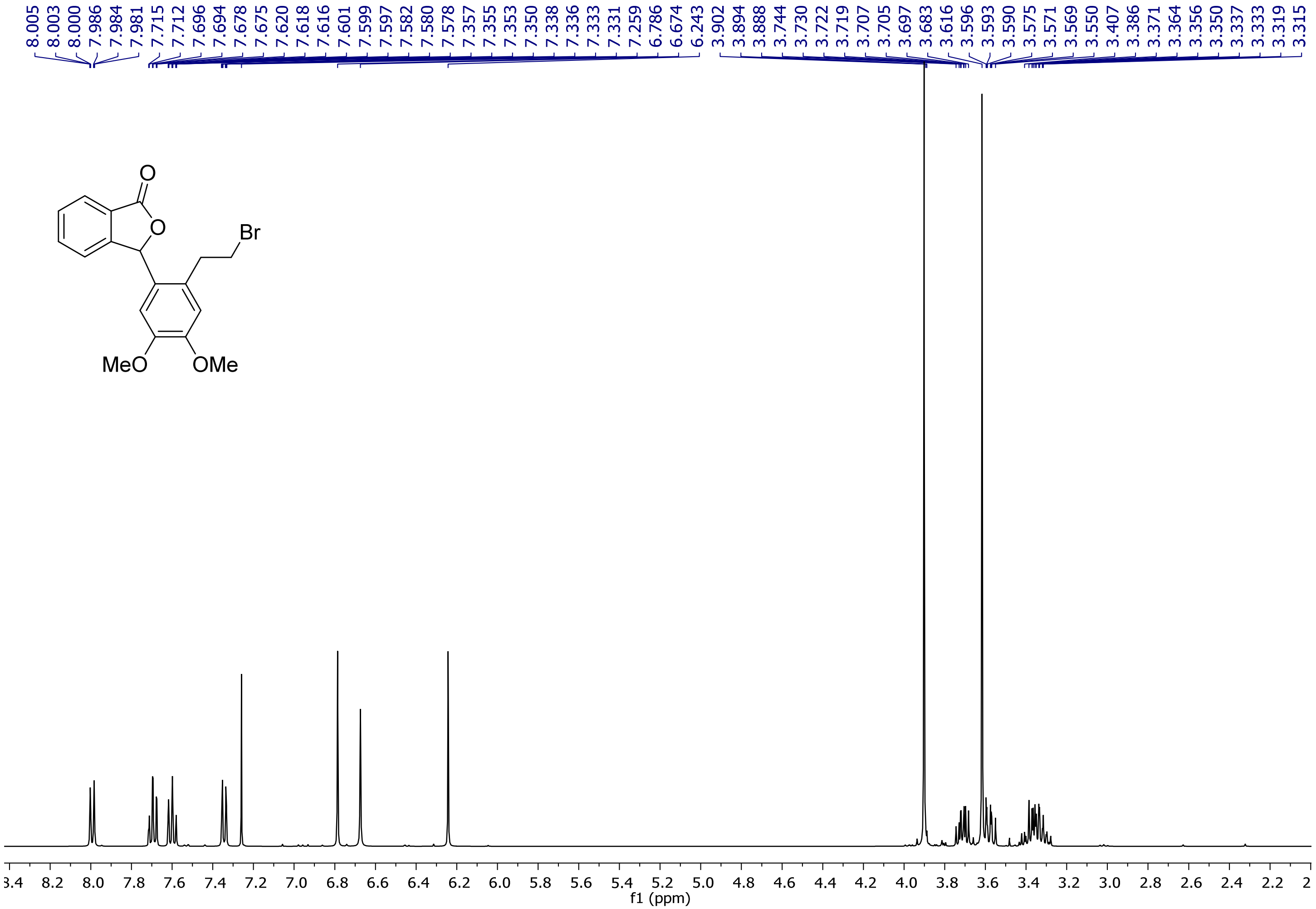


**Figure S5.** $^{1}$H NMR spectrum (400 MHz, $CDCl_3$) of compound **5c**

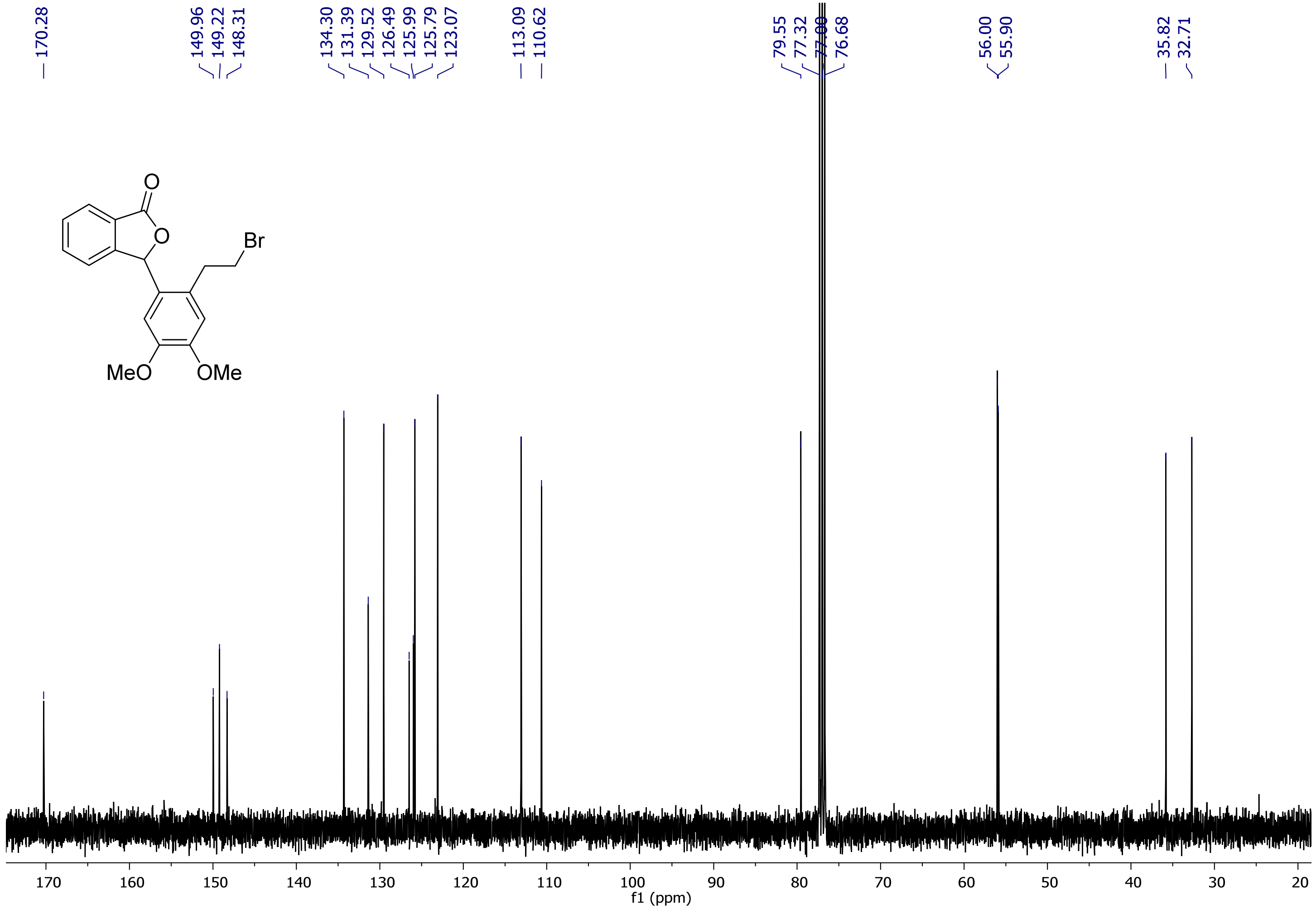


**Figure S6.** $^{13}C$ NMR spectrum (100 MHz, $CDCl_3$) of compound **5c**

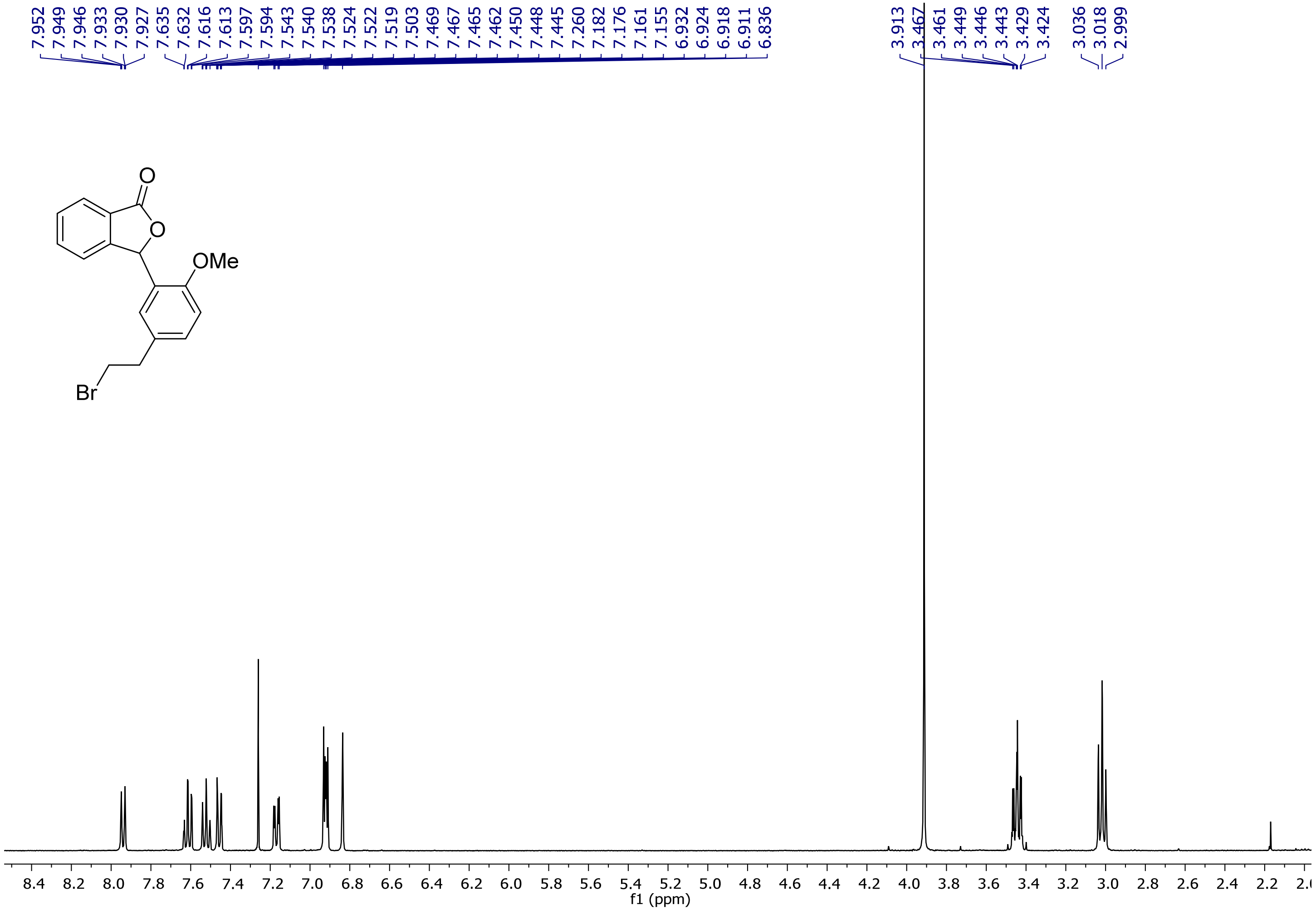


**Figure S7.** $^{1}H$ NMR spectrum (400 MHz, $CDCl_3$) of compound **5d**

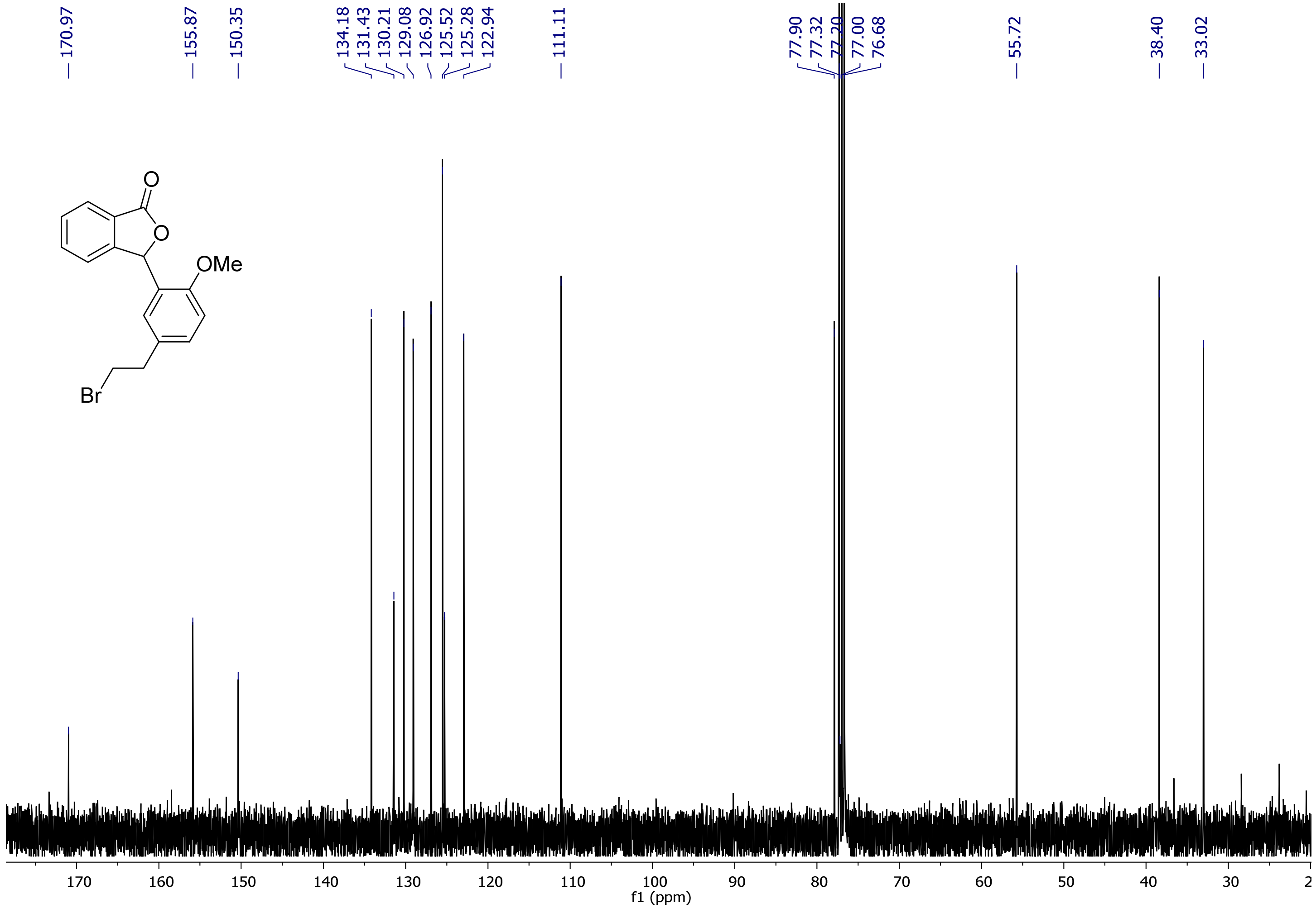


**Figure S8.** $^{13}C$ NMR spectrum (100 MHz, $CDCl_3$) of compound **5d**

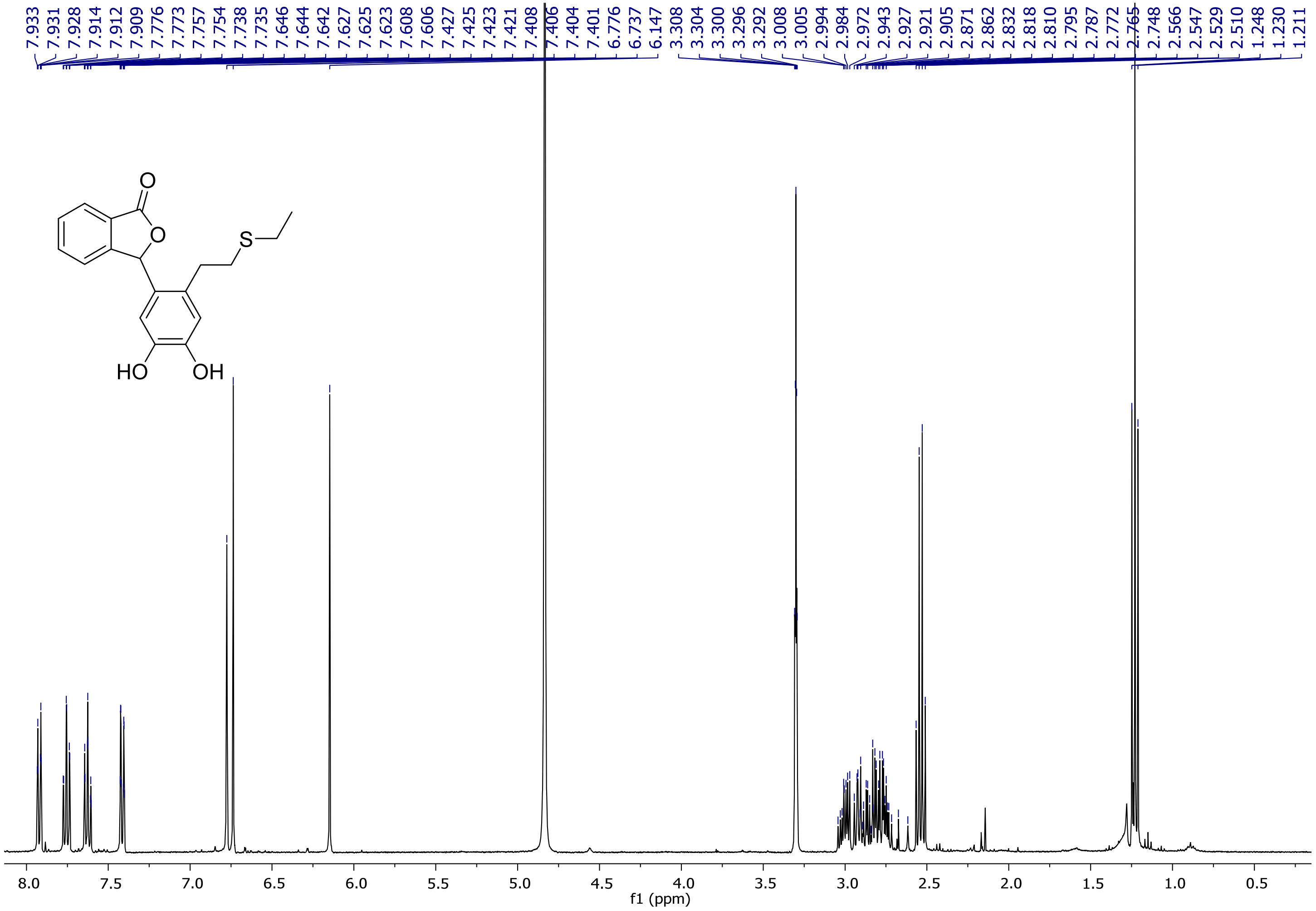


**Figure S9.** $^1$H NMR spectrum (400 MHz, $CD_3OD$) of compound **5e**

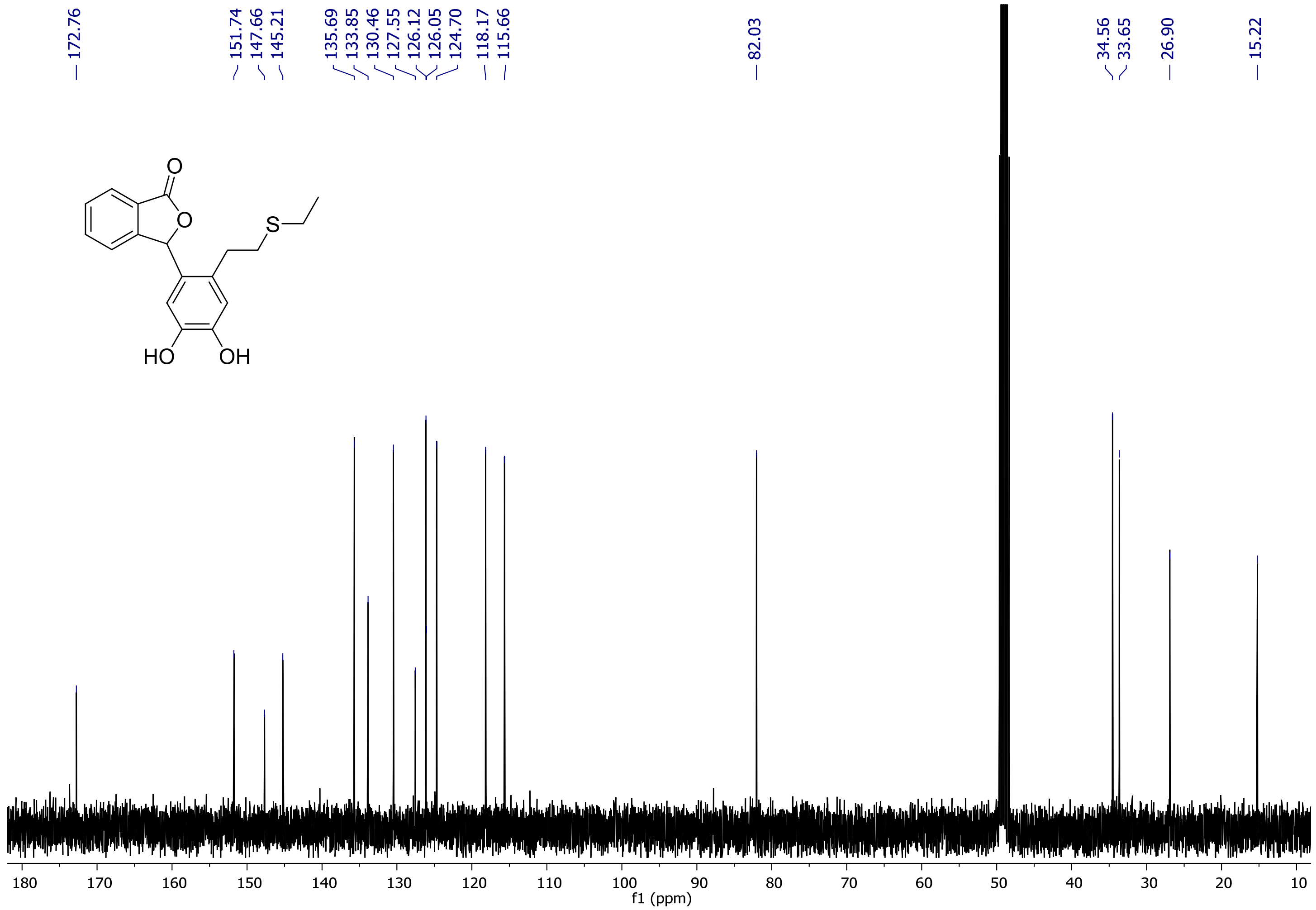


**Figure S10.** $^{13}$C NMR spectrum (100 MHz, $CD_3OD$) of compound **5e**

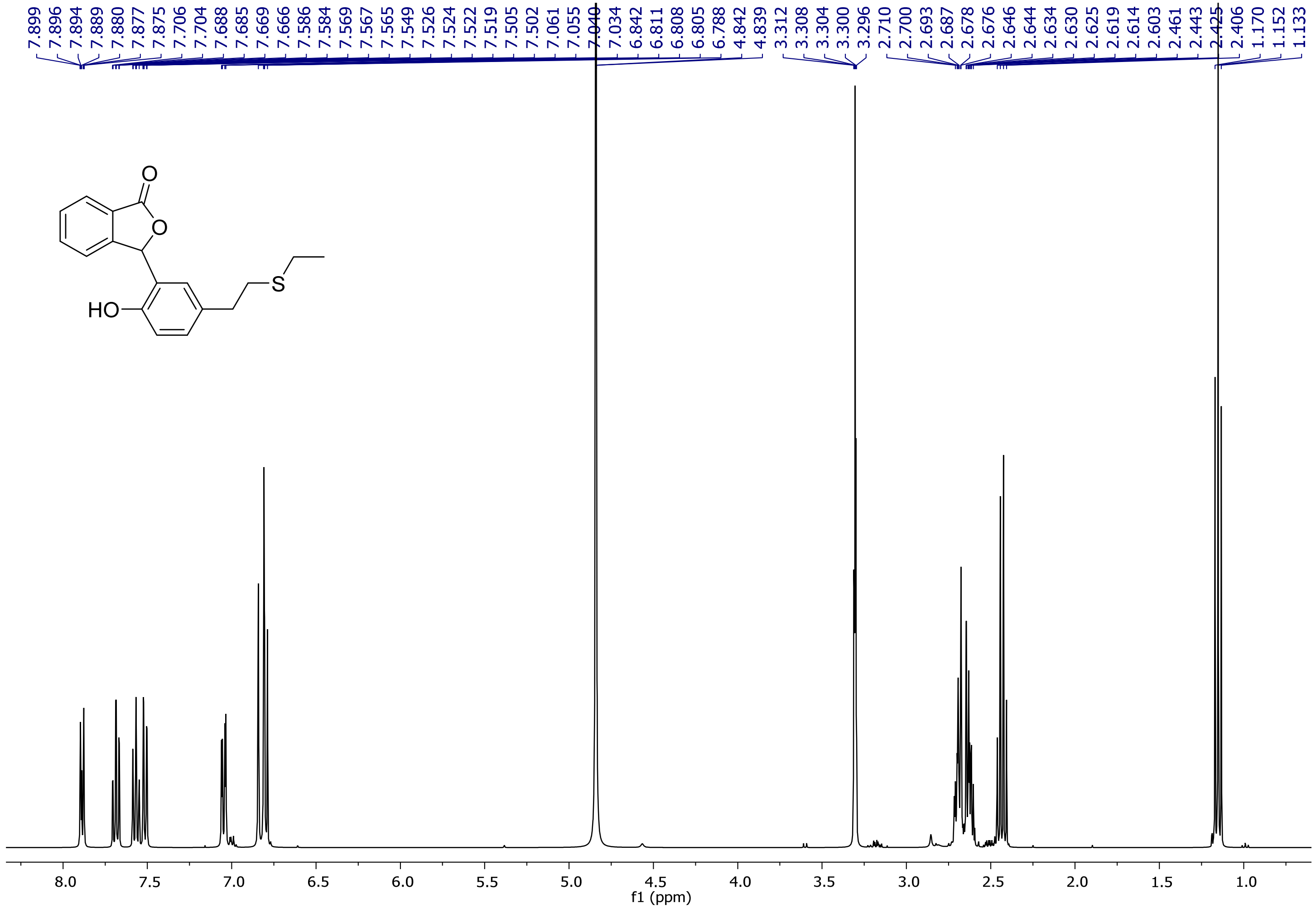


**Figure S11.** $^{13}$C NMR spectrum (400 MHz, $CD_3OD$) of compound **5f**

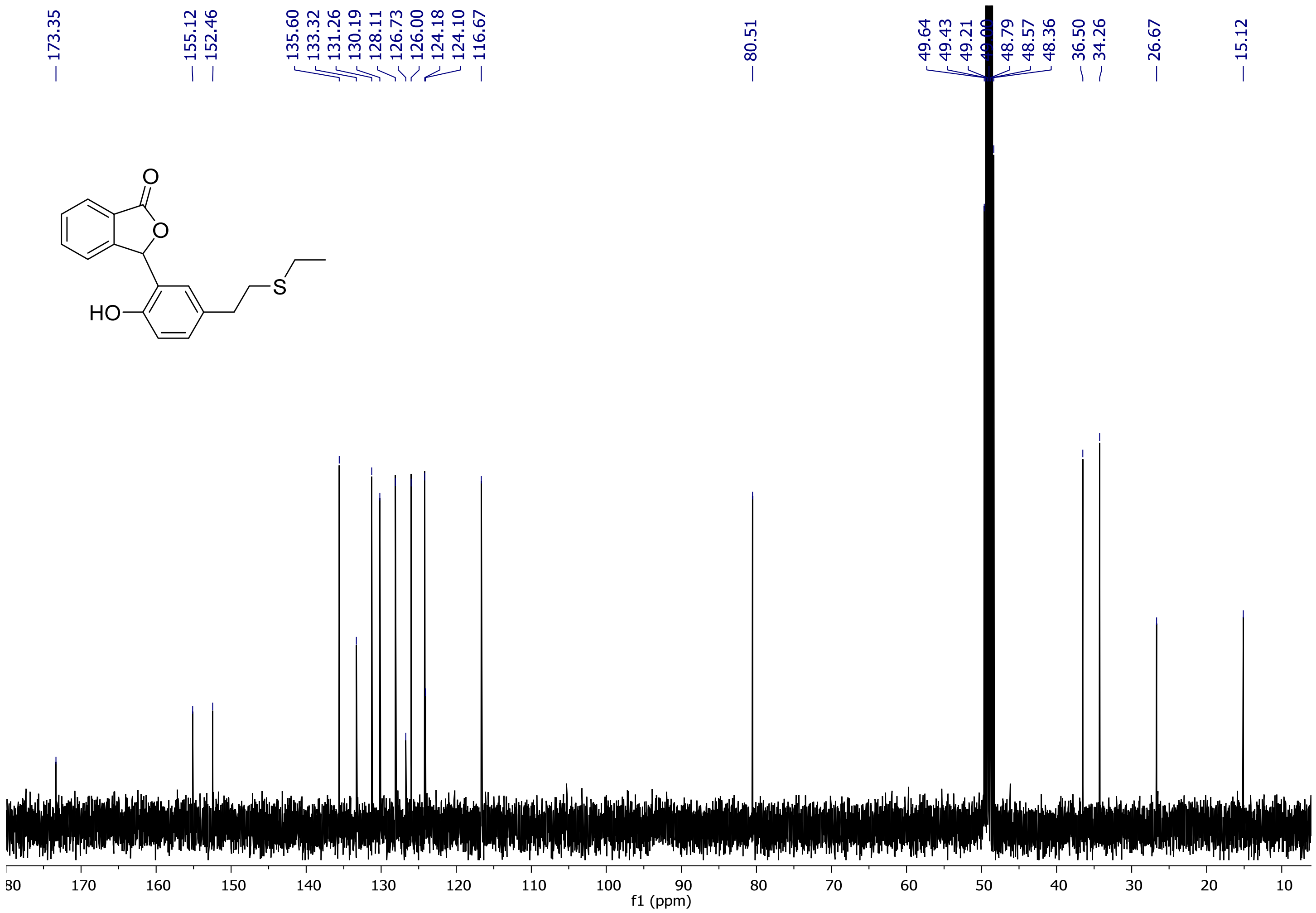


**Figure S12.** $^{13}C$ NMR spectrum (100 MHz, $CD_3OD$) of compound **5f**

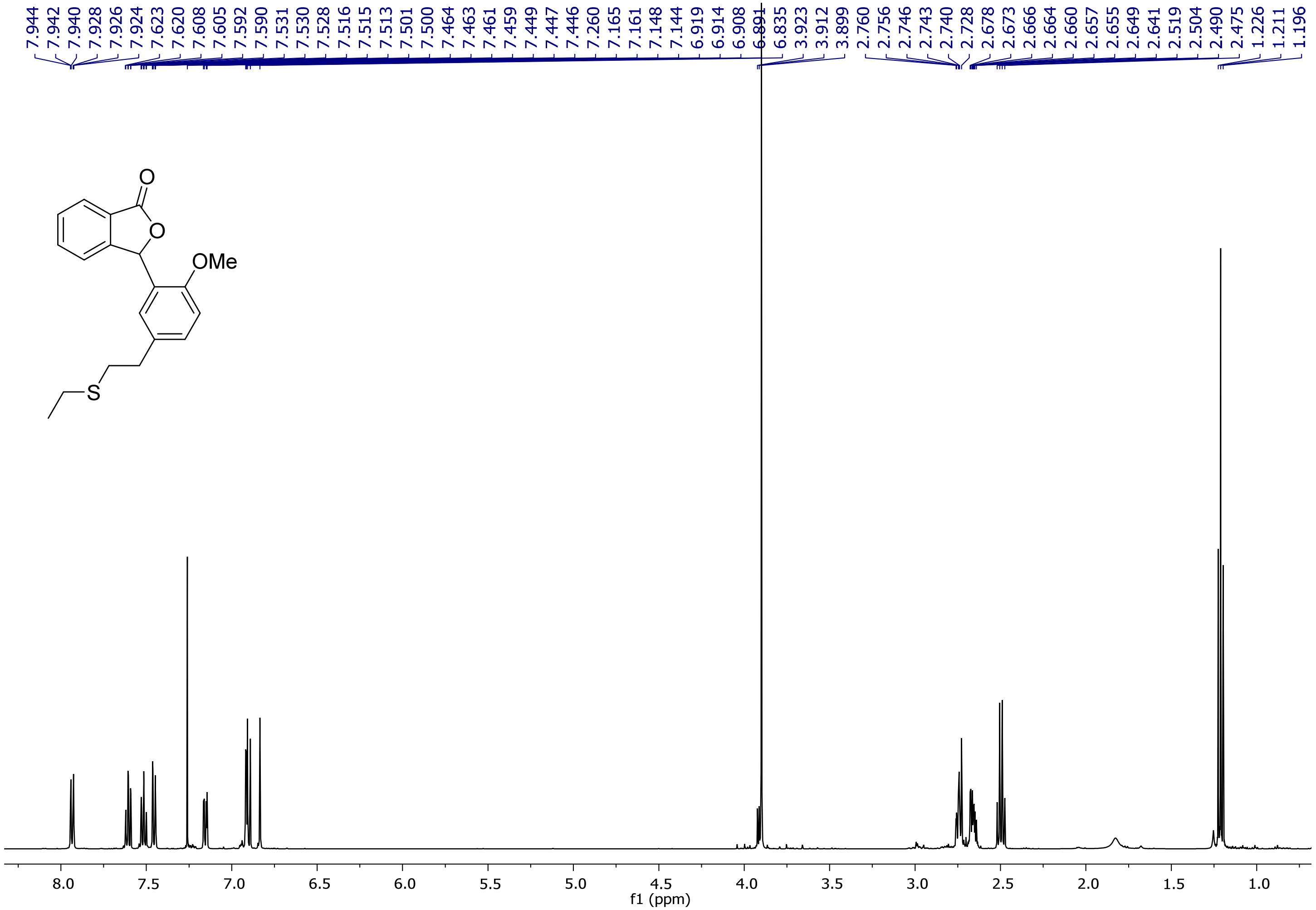


**Figure S13.** $^1$H NMR spectrum (500 MHz, $CDCl_3$) of compound **5g**

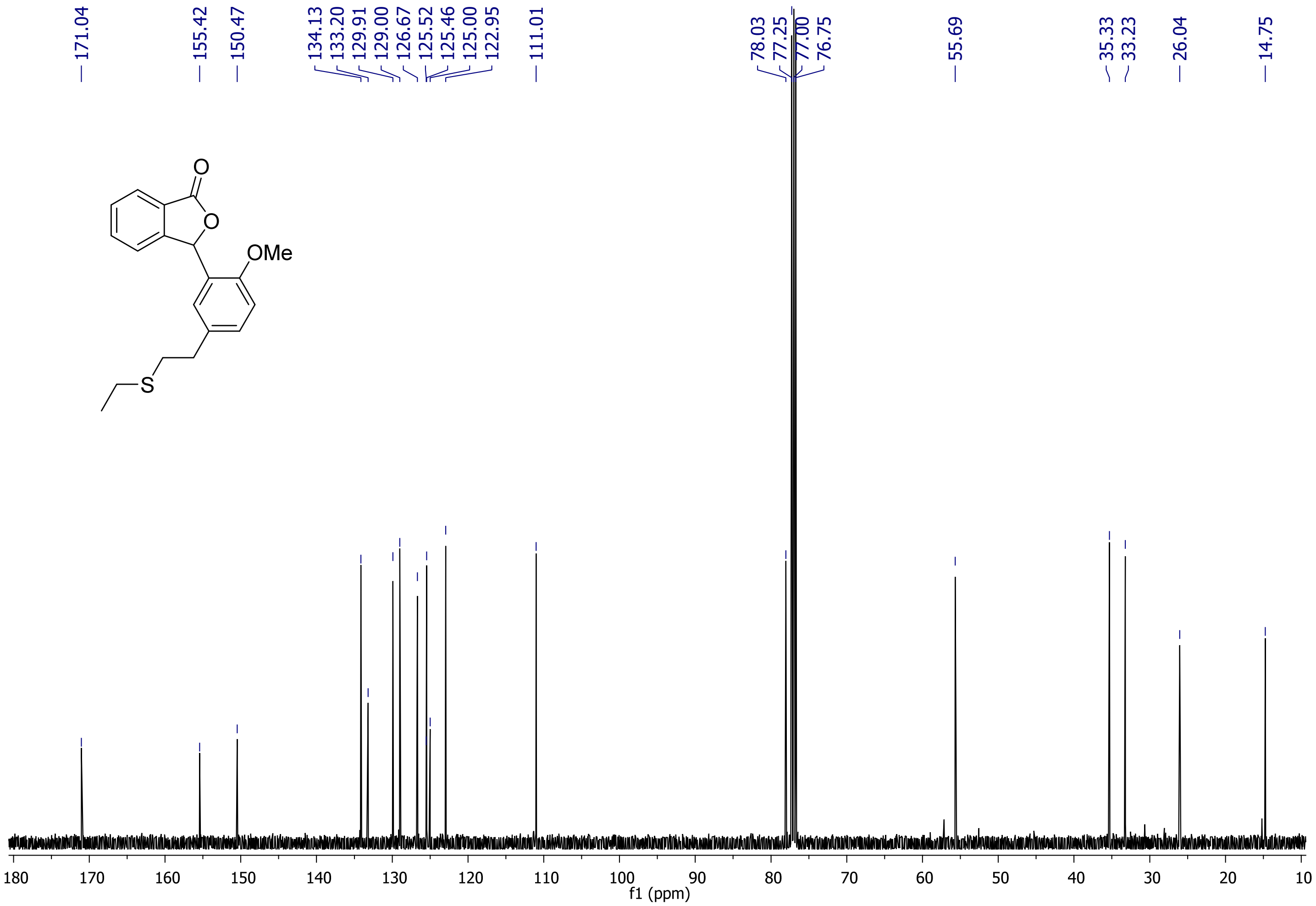


**Figure S14.** $^{13}$C NMR spectrum (125 MHz, $CDCl_3$) of compound **5g**

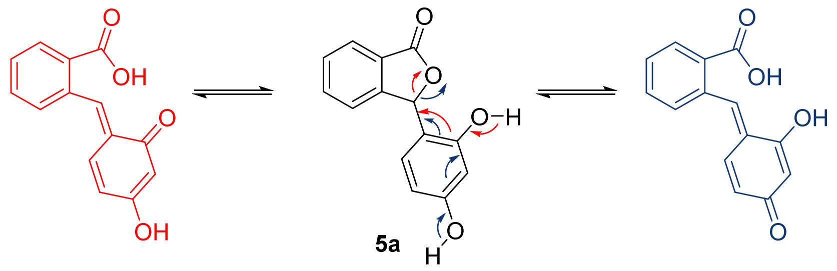


**Figure S15.** Proposed mechanism of racemization process in **5a**.

**S16**

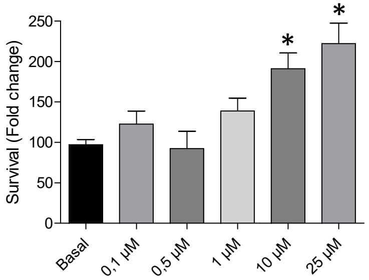


Compound **5a**

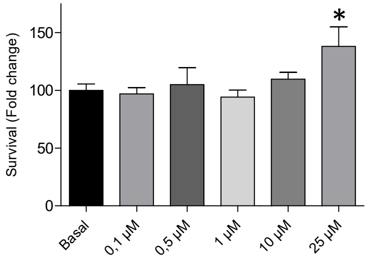


Compound **5b**

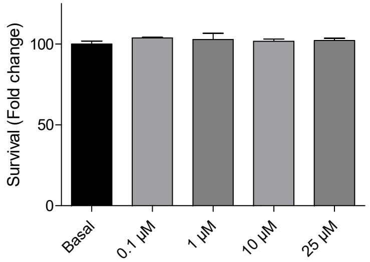


Compound **5c**

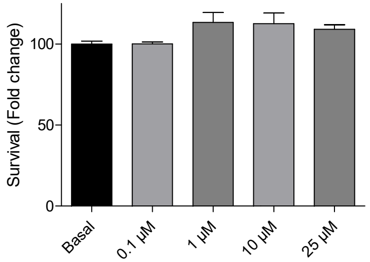


Compound **5d**

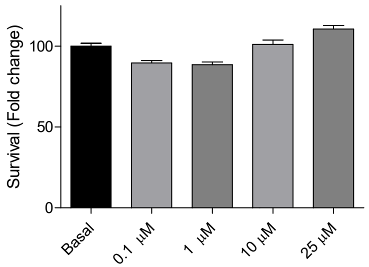


Compound **5e**

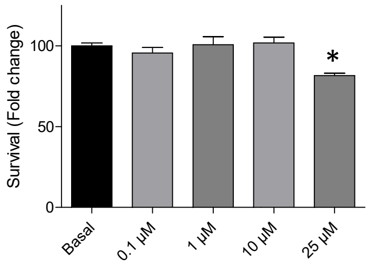


Compound **5f**

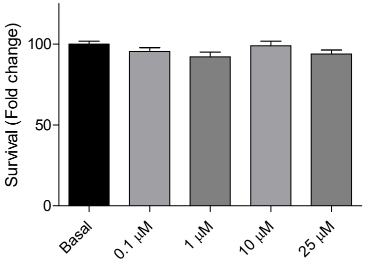


Compound **5g**

**Figure S16.** Dose-response cell viability of Bv.2 cells was determined by cristal violet assay. Colorimetric quantification was performed, and the results are mean ± SD (n ≥ 3 independent experiments performed in duplicate). Significant differences were determined by one way ANOVA followed by Bonferroni t-test *p ≤ 0.05 *vs* Basal.

**S17**

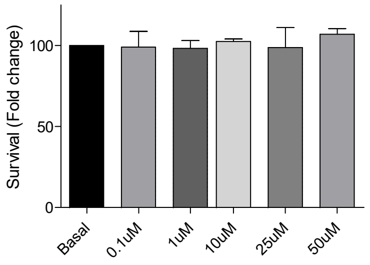


Compound **5a**

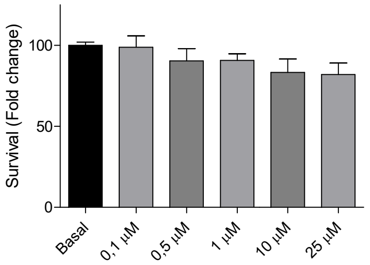


Compound **5b**

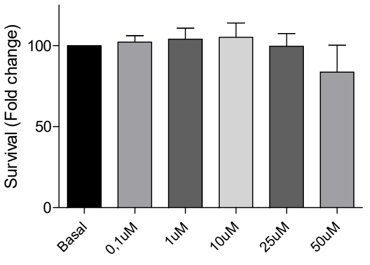


Compound **5c**

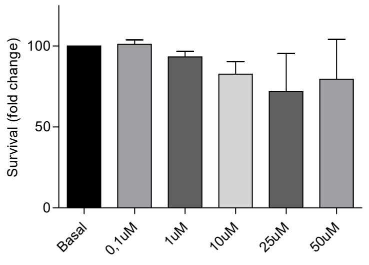


Compound **5d**

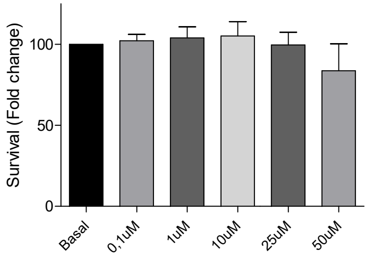


Compound **5e**

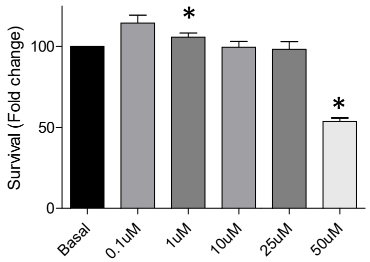


Compound **5f**

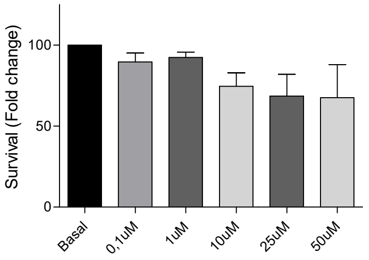


Compound **5g**

**Figure S17.** Dose-response cell viability of RAW 264.7 cells was determined by cristal violet assay. Colorimetric quantification was performed, and the results are mean ± SD (n ≥ 3 independent experiments performed in duplicate). Significant differences were determined by one way ANOVA followed by Bonferroni t-test *p ≤ 0.05 *vs* Basal.
